# Supplementary material for: Transnational conservation to anticipate future plant shifts in Europe
Source: Nat Ecol Evol. 2024 Jan 22;8(3):454–66. doi: 10.1038/s41559-023-02287-3 (PMC10927550; doi:10.1038/s41559-023-02287-3)
Supplement: Supplementary file 1 — Supplementary Methods 1–7, Figs. 1–38 and Tables 1–10. [file 41559_2023_2287_MOESM1_ESM.pdf]

nature ecology & evolution

Article

https://doi.org/10.1038/s41559-023-02287-3

Transnational conservation to anticipate future plant shifts in Europe

In the format provided by the authors and unedited

Appendix

Supplementary Methods 1. Methods details on the *Observations* section.

For (i), only observations accurate to 11.1 meters were kept. In total, 75 individual sources were compiled, including observational datasets from European research projects, institutions, research labs, universities, and monitoring programs, with largest contributions from the national data and information centre on the Swiss flora (InfoFlora, ~52%), and the French national alpine and Mediterranean botanical conservatory (CBNA/CNMED, ~7%; see Supplementary Table 2 for more information). To merge all datasets, we compiled a synonym list of all plant species (including orthographic variants) occurring in the European Alps, based on the Flora Alpina [1] backbone (~4490 accepted names). For this, a search was undertaken from different synonymy sources for each FA accepted name: Catalogue of life (http://www.catalogueoflife.org/), The plant List (http://www.theplantlist.org/), the French national alpine botanical conservatory (CBNA) and the Global Biodiversity Information Facility (GBIF; http://www.gbif.org/; using the *gbif.range* R package [2]). Although dealing with such a large amount of information may add discrepancy between sources (e.g., inconsistent, or duplicated synonyms), we removed synonyms if disagreements were found to obtain a total of 131.660 unique synonyms. Finally, all datasets were merged and harmonized into one by undertaking name matching and manual checks.

For (ii), our 131.660 synonyms were used as search inputs in GBIF to retrieve online observations using the *gbif.range* R package [2]. Only observations with a 100% confidence name matching and accurate to 11.1 meters were kept. GBIF provides a huge amount of georeferenced species distribution data, but many observations have considerable coordinate uncertainties, duplicated records, or misleading raster centroids. Therefore, strong spatial and resolution filtering needed to be applied to the online dataset to make sure that no biased species observations would be used in our models. The filtering involved mainly: selection based on GBIF's basis of records ('HUMAN_OBSERVATION', 'LITERATURE', 'MATERIAL_SAMPLE', 'OBSERVATION', 'MATERIAL CITATION' kept), removal of observation duplicates, removal of observations without coordinates, removal of absence records, removal of observations with equal latitude and longitude, removal of observations identified as having corrupted coordinates, removal of observations older than 1970, removal of coordinates with less than four decimals, and removal of raster centroid datasets [3].

Supplementary Methods 2. Full methods of the *Soil* section

We derived soil property layers at a 100 m resolution over the study area by mapping ecological indicator values (EIVs)[4,5] in space following the method described in [4]. First, we obtained plant EIVs from Flora Alpina[1] and retained two different EIVs to characterize the local edaphic conditions: soil nitrogen (EIV-N) and soil substrate composition (EIV-G). EIVs of the Flora Alpina are ordinal variables consisting of three classes each (low = 1, medium = 2, high = 3) and associated estimated species frequency in the classes (not occurring = 0, rare = 0.5, frequent = 1). For each species, we calculated the average EIV value weighted by the frequency of the species in the EIV classes. Second, EIV values were combined to our unfiltered observational dataset (i.e., 6'655'163 observations of 4'250 plant species) mapped on a 100 m grid. We removed duplicate observations of individual species per grid cell. Grid cells including at least ten species were then kept, to calculate for each EIV a mean value across all species' EIV values per grid cell. EIV models were then calibrated from these cells at 100 m spatial resolution with Random Forest[6] using variables representing topography and geology (see Supplementary Table 4 for the list of variables), and were predicted across the study area at a 100 m grain. EIV models were evaluated using Spearman's rank correlation tests by partitioning the data into training (80%) and evaluation (20%) sets following two approaches: (i) five-fold split-sampling of the data and (ii) five-fold spatial block cross-validation [7] using five strata assigned across 25 regional clusters across the Alps. All generated EIV soil property layers showed excellent evaluations with Spearman $r > 0.82$ (see Supplementary Table 5). The generated EIV soil property layers are proxies of soil nitrogen (NITROGEN) and substrate composition (CALCAREOUS%), and have been shown to be excellent predictors of plant species distribution in SDMs [4,8]. It is important to note that given the unavailability of future predicted soil information, we considered current and future soil unchanged.

Supplementary Methods 3. Full methods of the *EBC* section

Before data collection, appropriate sampling design should be environmentally stratified [9–12]. Sampling frequencies in environmental space may in fact still remain skewed if species observations are not initially sampled according to an environmental stratification. Therefore to additionally address the environmental bias in the sampling design of our refined observational dataset (see Supplementary Figure 35), a recent corrective method, based on environmental stratified resampling of the observational dataset, was implemented before model calibration using the R function *wsl.ebc* [13]. Environmental bias correction (EBC) corrects potential environmental bias in the design of an observational dataset, by artificially sub-sampling original species observations based on a chosen number of environmental clusters over the study area. Using the R function clValid (R package clValid) [14] and following recommendations of [13], we set the number of environmental clusters to 20, i.e. summarizing not too precisely the environmental space of the study area, yet, large enough to account for the its environmental complexity. Within the function *wsl.ebc*, we set the different parameters as follows: (i) ras = the four current climate and two soil predictors (same as in SDM calibration); (ii) pportional = TRUE, i.e. to apply EBC with a proportional stratified sampling design (or EBCp); (iii) plog = TRUE, i.e. EBCp should adopt a log consensus; (iv) sp.specific = TRUE, i.e. EBCp applies only for species subject to an environmental bias; (v) sp.cor = 0.5, i.e. correlation threshold of the environmental bias; (vi) keep.bias = TRUE, i.e. per species and after EBC applies, the number of observations in the densest original cluster is reset to that of the densest corrected cluster; (vii) filter = FALSE; i.e. the observations are not filtered according to the ras resolution. In total, EBC applied to 1’248 species. The resulting corrected observations and their environmental frequencies (before and after EBC) may be found in Supplementary Figure 36.

Supplementary Methods 4. Full methods of the SDM *Calibration* section

For each species, model calibrations were done at 100 m resolution, by including current climate/LC (1 km), soil (100 m) as well as our bias predictors (100 m), and were calibrated twice i.e., one model per categorical LC. We used a special case of presence-only SDM, namely point-process models (PPMs), whose output represents the intensity of the expected number of species occurrences per unit area, that is modelled as a log-linear function of the environmental covariates[15,16]. Although described as an equivalent of MAXENT [17], PPMs have many more methodological benefits [13,15,16]. First, unlike most SDM approaches [18,19], PPM propose an automated framework to choose the number and location of 'quadrature points' (commonly referred to as 'background points' or 'pseudo-absences') [15], if no true absences are available. Second, on top of dealing with observer bias more objectively [13] (see *Bias covariate correction* section above), PPM indirectly avoids incomplete species response curves thanks to the adequate number of quadrature points automatically sampled across the whole environmental gradient [15,16]. Finally, PPM may be easily employed along lasso and clarifies the form of the modelled response as it represents an intensity of species observation (or abundance) and not a probability [15,17]. At location s, the intensity (s) is thus given by:

$$\ln\lambda(\mathrm{s}) = \mathbf{x}(\mathrm{s})'\boldsymbol{\beta} \qquad \text{[eqn 2]}$$

where $\boldsymbol{x}(s)$ is the vector of the p environmental covariates, and $\boldsymbol{\beta} = \{\beta_1, \ldots, \beta_p\}$ is the vector of the corresponding regression coefficients. Given the vector of n observations $\boldsymbol{s}_P = \{s_1, \ldots, s_n\}$, PPMs are fitted via maximum likelihood, and the parameters are found to maximise [20]:

$$l(\boldsymbol{\beta};\ \boldsymbol{s}_P) = \sum_{i=1}^{n} \ln\lambda(s_i)\ -\ \int_A \lambda(s)\,ds \qquad \text{[eqn 3]}$$

where the integral of the intensity over the entire study area **A** has to be approximated numerically. Such approximation requires the introduction of 'quadrature points, a set of points at which the intensity function is evaluated. As discussed in detail in [15], these points are needed to approximate the integral. The likelihood can thus be approximated as:

$$l(\boldsymbol{\beta};\ \boldsymbol{s}_P) \approx \sum_{i=1}^{n} \ln\lambda(s_i) - \sum_{j=1}^{n+m} w_j\lambda(s_j) \qquad \text{[eqn 4]}$$

$$= \sum_{i=1}^{n+m} w_i\ (y_i \ln\lambda(s_i) - \lambda(s_i)\ ) \qquad \text{[eqn 5]}$$

Where $\boldsymbol{s}_0 = \{s_{n+1}, \ldots, s_{n+m}\}$ are the quadrature points and $\boldsymbol{w} = \{w_1, \ldots, w_{n+m}\}$ the quadrature weights. Since this is the likelihood of a weighted Poisson regression, PPMs belong to the family of generalized linear models (GLMs) [21] and can be fitted using any package in **R** capable of fitting GLMs. We therefore used the basic function *glm*, and we calibrated our models as a 'down-weighted Poisson regression' (DWPR) [15] with second-order polynomials (except for categorical land cover), and elastic net regularization [22] for all covariates.

DWPR with Elastic Net was executed using the R package *glmnet* [23,24]. Elastic Net represents a type of regularization and variable selection mixing lasso and ridge regression approaches [22]. It penalizes non-relevant predictors that might lead to overfitting by shrinking their effects or removing them completely. Elastic net regularization requires two parameters: alpha (α), which sets the balance between lasso and ridge, and lambda (λ), which sets the penalty coefficient level. For model, we set α to 0.5, and determined the optimal λ by testing 100 different values and selecting the one for which model fit performed best under a new 5-fold cross-validation (function *cv.glmnet*).

Quadrature points were sampled randomly without replacement across the study area over a 100 m regular mesh. Because log-likelihood convergence is model specific, we estimated for each PPM the appropriate number of quadrature points by running 10 repeated series of DWPR and gradually increasing the number of randomly sampled points from 5000 to 600'000 points following [15] (see Supplementary Figure 37). For weights implemented in model calibrations, small ones were assigned to observations (1e-06), and given a study area of 294'994 $km^{2,}$ quadrature weights (*QW*) were calculated following [15]:

$$QW = study\ area\ (km^2)/quadrature\ points\ (n) \quad \text{[eqn 6]}$$

Therefore, when n = 5000, QW ≈ 589.9; n = 10’000, QW ≈ 29.5; n = 20’000, QW ≈ 14.7; n = 50’000, QW ≈ 5.9; n = 100’000, QW ≈ 2.9; n = 150’000, QW ≈ 1.9; n = 200’000, QW ≈ 1.5; n = 250’000, QW ≈ 1.2; n = 400’000, QW ≈ 0.7; n = 600’000, QW ≈ 0.5.

Supplementary Methods 5. Methods details on the *Diversity* section

Taxonomic. For each 13 scenarios, we generated a community matrix summarizing the absolute occurrence intensities of the 1'711 species per pixel across the study area. For each matrix, TD was calculated as abundance-based species diversity based on Hill numbers [25] with the R package *hillR* (function *hill_taxa*) [26]. Here, we chose the order q = 1 (or the Shannon entropy ***H***), i.e., an average sensitivity of the calculated pixel diversity to species absolute occurrence intensities.

Phylogenetic. Based on the detailed name list of our 1'711 modelled species (family, genus and species), a phylogenetic tree was computed using the R package *V.PhyloMaker* [27] (function *phylo.maker*). *V.PhyloMaker* may generate large phylogenies for vascular plants based on updated versions of two plant mega-phylogenies [28,29]. These phylogenies were built based on fossil records, molecular data from GenBank (https://www.ncbi.nlm.nih.gov/genbank/) and phylogenetic data from the Open Tree of Life (https://tree.opentreeoflife.org/), including over 70 000 species of vascular plants. Using the phylogeny, PD was calculated for each community matrix based on extended Hill numbers to phylogenetic diversity (function *hill_phylo*) [26,27]. Same Hill order was here kept (q =1), i.e. phylogenetic diversity of each pixel was here calculated with the phylogenetic entropy $\boldsymbol{H}_{\mathrm{p}}$; a generalization of the Shannon entropy [30,31]. It is generally known that PD is not independent from TD [32,33]. Therefore, the residuals of a linear regression of TD on PD (quadratic terms included) were extracted to generate new layers of relative phylogenetic diversity (rPD) [34–36].

Functional. (i) Trait data was compiled from 33 individual sources comprising national data centres (e.g., InfoFlora, CBNA), European projects (e.g., OriginAlps, Fifth, Ecochange) and various sources from the literature and from collaborators (for a complete list, see Supplementary Table 8). Mean plant height, leaf dry matter content, specific leaf area and leaf carbon to nitrogen ratio were extracted for our 1'711 species (i.e., 4'308 traits). In total, 1'344 species were missing at least one trait (~35% of total missing values). (ii) Missing information in trait databases is a chronic issue in ecological studies [37,38]. While removing species with absent information is considered a common practice [39,40], such practice is problematic if data are not missing taxonomically or phylogenetically at random (MCAR) [40,41]. An alternative imputation is preferred and strongly recommended when trait data are indeed missing at random (MAR) [39,40]. Our trait data had ~35% of missing values (2'536 measurements out of 6'844); i.e. an acceptable percentage under both MCAR and MAR assumptions [39,42]. We tested both assumptions by applying preliminary MCAR (*MissMech* R package) [43] and multiple-imputation tests (R package *mice*) [44] respectively, and found our missing data to follow rather the MAR than the MCAR assumption (see Supplementary Figure 38). We therefore substituted missing trait values by implementing the MAR imputation method (function *mice*, method *rf)*, which has demonstrated good performances of trait data imputation [39,40]. (iii) Trait values were normalized from 0 to 1 and Gower's distances were calculated thereof [45,46]. Based on the distance matrix, functional dendrograms were generated from different algorithms with the R package *cluster* (function *daisy)* [47]. We kept the functional tree whose cophenetic distance matrix was the most correlated with the initial distance matrix (UPGMA functional tree) [48]. Finally, based on a Mantel test using 9'999 randomizations (R package *vegan*, function *mantel*) [49], we found the functional dendrogram to express ~78% of the initial distance matrix (r = 0.777; *** *P-value* < 0.001) indicating a strong conservation of the original functional space. (iv) Using the obtained functional dendrogram, FD was calculated for each community matrix based on extended Hill numbers to functional diversity (function *hill_func*) [26,50]. Same Hill order was here kept (q =1), i.e. functional diversity of each pixel was here calculated with a compromise index, i.e. at the interface between the functional attribute diversity and the weighted Gini-Simpson index [30,51,52]. As PD, FD is generally not independent from TD. Therefore, the residuals of a linear regression of TD on FD (quadratic terms included) were also extracted to generate new layers of relative functional diversity (rFD).

Supplementary Methods 6. Methods details on the *Uniqueness* section

Using our phylogeny and functional dendrograms three types of endemism were calculated for each community matrix. (1) Weighted taxonomic endemism ***WE*** is defined as the species richness in one pixel divided by the sum of the species ranges [53]:

$$\mathrm{WE} = \sum_{t \in T} \frac{1}{R_t} \quad \text{[eqn 7]}$$

where $\boldsymbol{R_t}$ represents the regional geographic range of species ***t*** (or number of pixels over the study area where its occurrence intensities > 0). WE was calculated with the R package *phyloregion* (function *weighted_endemism)* [54]. (2) Weighted phylogenetic endemism ***PE*** is defined as the sum of branch length in one pixel divided by the regional range of each branch on the spanning path connecting a set of taxa to the root of a phylogenetic tree [55]:

$$\mathrm{PE} = \sum_{c \in C} \frac{L_c}{R_c} \quad \text{[eqn 8]}$$

where $\boldsymbol{R_c}$ represents the regional geographic range of branch ***c*** (or number of pixels over the study area where the branch occurs), and $\boldsymbol{L_c}$ the length of branch ***c***. PE was calculated with the R package *phyloregion* (function *phylo_endemism)* [54]. (3) Weighted functional endemism ***FE*** was calculated based on our functional dendrogram with the same approach as PE:

$$\mathrm{FE} = \sum_{i \in I} \frac{L_i}{R_i} \quad \text{[eqn 9]}$$

where $\boldsymbol{R_i}$ represents the regional geographic range of the functional branch ***i*** (or number of pixels over the study area where the branch occurs), and $\boldsymbol{L_i}$ the length of functional branch ***i***. FE was calculated with the R package *phyloregion* (function *phylo_endemism)* [54]. Relative phylogenetic and functional endemism (rPE and rFE) were generated following the same procedure and justifications as for rPD and rFD. Finally, we also calculated for each scenario phylogenetic (PR) and functional rarity (FR) across the study area following [56]. Using our functional dendrogram, functional rarity (***FR***) [57] was calculated for each community matrix (preliminary converted to relative abundance matrix as specified by [56] based on four distinct metrics using the R package *funrar* (function *funrar*) [56]: (1) scarcity, (2) geographical restrictiveness, (3) functional distinctiveness and (4) uniqueness. (1) Scarcity ($\boldsymbol{S_t}$) defines the local rarity of a species:

$$S_t = \exp\left(-NA_t \ln(2)\right) \quad \text{[eqn 10]}$$

where $\boldsymbol{N}$ is the number of species and $\boldsymbol{A_t}$ the occurrence intensity of species ***t*** in one pixel. (2) Restrictiveness ($\boldsymbol{GR_t}$) defines the regional rarity of a species:

$$GR_t = 1 - \frac{R_t}{R_{total}} \quad \text{[eqn 11]}$$

where $\boldsymbol{R_t}$ is the number of pixels where species ***t*** occurs and $\boldsymbol{R_{total}}$ the total number of pixels of the study area. (3) Functional distinctiveness ($\boldsymbol{D_t}$) defines the uncommonness of traits of a species compared to other species' traits in one pixel weighted by their absolute occurrence intensities:

$$D_t = \frac{\sum_{j=1, j\neq t}^{N} d_{tj} A_j}{\sum_{j=1, j\neq t}^{N} A_j} \quad \text{[eqn 12]}$$

where $\boldsymbol{d_{tj}}$ is the functional dissimilarity between species ***t*** and species ***j***, ***N*** and $\boldsymbol{A_j}$ the number of species and the absolute occurrence intensity of species ***j*** in the pixel respectively. (4) Functional uniqueness ($\boldsymbol{U_t}$) is the functional distance of a species ***t*** to its closest neighbour in a given region:

$$U_t = \min(d_{tj}) \qquad \text{[eqn 13]}$$

where $\boldsymbol{d}_{tj}$ is the functional dissimilarity between species $\boldsymbol{t}$ and species $\boldsymbol{j}$. Scarcity, restrictiveness, functional distinctiveness, and uniqueness were calculated for each species and pixel. Unlike scarcity and distinctiveness, restrictiveness and uniqueness are only calculated per species. Therefore, to obtain values per species and pixel, each species' relative abundances across the study area were weighted by its level of restrictiveness and uniqueness respectively. Total scarcity ($\boldsymbol{S}_{total}$), restrictiveness ($\boldsymbol{GR}_{total}$), functional distinctiveness ($\boldsymbol{D}_{total}$) and uniqueness ($\boldsymbol{U}_{total}$) were each obtained over the study area by averaging values across species. As suggested by [56], each spatial metric were normalized from 0 to 1 and functional rarity was obtained by calculating the average:

$$FR = \frac{S_{total} + GR_{total} + D_{total} + U_{total}}{4} \qquad \text{[eqn 14]}$$

Finally, using the same relative abundance matrices and our phylogeny, 13 phylogenetic rarity (***PR***) [58] were calculated over the study region with the same approach as functional rarity using the R package *funrar* (function *funrar*) [56].

Supplementary Methods 7. Methods details on the *Post-analyses* section

For this purpose, aggregated SDM occurrence intensities of each species were employed and each phylogenetic and functional branch's spatial occurrence intensities were calculated following [59]. While the occurrence intensity of terminal branches is inferred from that of the species, the occurrence intensity of internal branches in each cell is calculated as:

$$B_{ij} = 1 - \prod_{n-1}^{m}(1 - P_{nj}) \qquad \text{[eqn 15]}$$

where $\boldsymbol{B}_{ij}$ is the intensity of an internal branch ***i*** in cell ***j***, ***m*** the number of descendant species originating from the internal branch and $\boldsymbol{P}_{nj}$ the intensity of descendent species ***n*** to occur in cell ***j***. Therefore, for each percentage of expanding PAs over the study region, we calculated the cumulative representation of (1) species, (2) phylogenetic and (3) functional branch occurrence intensities, of (4) species, (5) phylogenetic and (6) functional branch distributional range, and of species (7) functional and (8) phylogenetic rarity. (2) The cumulative representation of phylogenetic diversity (PD) was calculated following [59]:

$$PD\ (\%) = \frac{1}{\sum_{i=1}^{k} L_i} \cdot \sum_{i=1}^{k} \left[\left(L_i \cdot \frac{\sum_{j=1}^{q} B_{ij}}{\sum_{j=1}^{Q} B_{ij}} \right) \right] \times 100 \qquad \text{[eqn 16]}$$

where ***k*** is the number of branches of the phylogenetic tree, ***q*** the cells covered by PAs (%), ***Q*** the total number of cells in the landscape, $\boldsymbol{B}_{ij}$ the occurrence intensity of branch ***i*** in cell ***j*** and ***L*** is the length of branch ***i***. (3) The cumulative representation of functional diversity (FD) was calculated the same way but using the functional branches information. (1) Based on the previous equation, the cumulative representation of species diversity (TD) was calculated as:

$$TD\ (\%) = \frac{1}{N} \cdot \sum_{i=1}^{N} \left[\frac{\sum_{j=1}^{q} A_{ij}}{\sum_{j=1}^{Q} A_{ij}} \right] \times 100 \qquad \text{[eqn 17]}$$

where ***N*** is the total number of species, ***q*** the cells covered by PAs (%), ***Q*** the total number of cells in the landscape, $\boldsymbol{A}_{ij}$ the occurrence intensity of species ***i*** in cell ***j***. (4) The cumulative representation of species endemism (WE) was calculated following [60]:

$$WE\ (\%) = \frac{1}{N} \cdot \sum_{i=1}^{N} \frac{s_i}{S_i} \times 100 \qquad \text{[eqn 18]}$$

where ***N*** is the total number of species, $\boldsymbol{s}_i$ the number of cells covered by PAs (%) where the species occurs, and $\boldsymbol{S}_i$ the total number of cells where the species occurs. (5) The cumulative representation of phylogenetic endemism (PE) was calculated following [60]:

$$PE\ (\%) = \frac{1}{\sum_{i=1}^{k} L_i} \cdot \sum_{i=1}^{k} \left[L_i \cdot \frac{e_i}{E_i} \right] \times 100 \qquad \text{[eqn 19]}$$

where ***k*** is the number of branches of the tree, $\boldsymbol{e}_i$ the number of cells covered by PAs (%) where the branch occurs, $\boldsymbol{E}_i$ the total number of cells where the branch occurs, and ***L*** is the length of branch ***i***. (6) The cumulative representation of functional endemism (FE) was calculated the same way but using the functional branches information. (7) The cumulative representation of species functional rarity (FR) was adapted from [eqn 17] and calculated as:

$$FR\ (\%) = \frac{1}{N} \cdot \sum_{i=1}^{N} \left[\frac{\sum_{j=1}^{q} R_{ij}}{\sum_{j=1}^{Q} R_{ij}} \right] \times 100 \qquad \text{[eqn 20]}$$

where ***N*** is the total number of species, ***q*** the cells covered by PAs (%), ***Q*** the total number of cells in the landscape, and $\boldsymbol{R}_{ij}$ the functional rarity of species ***i*** in cell ***j***. (8) The cumulative representation of species phylogenetic rarity (PR) was calculated the same way but using the phylogenetic rarity of each species.

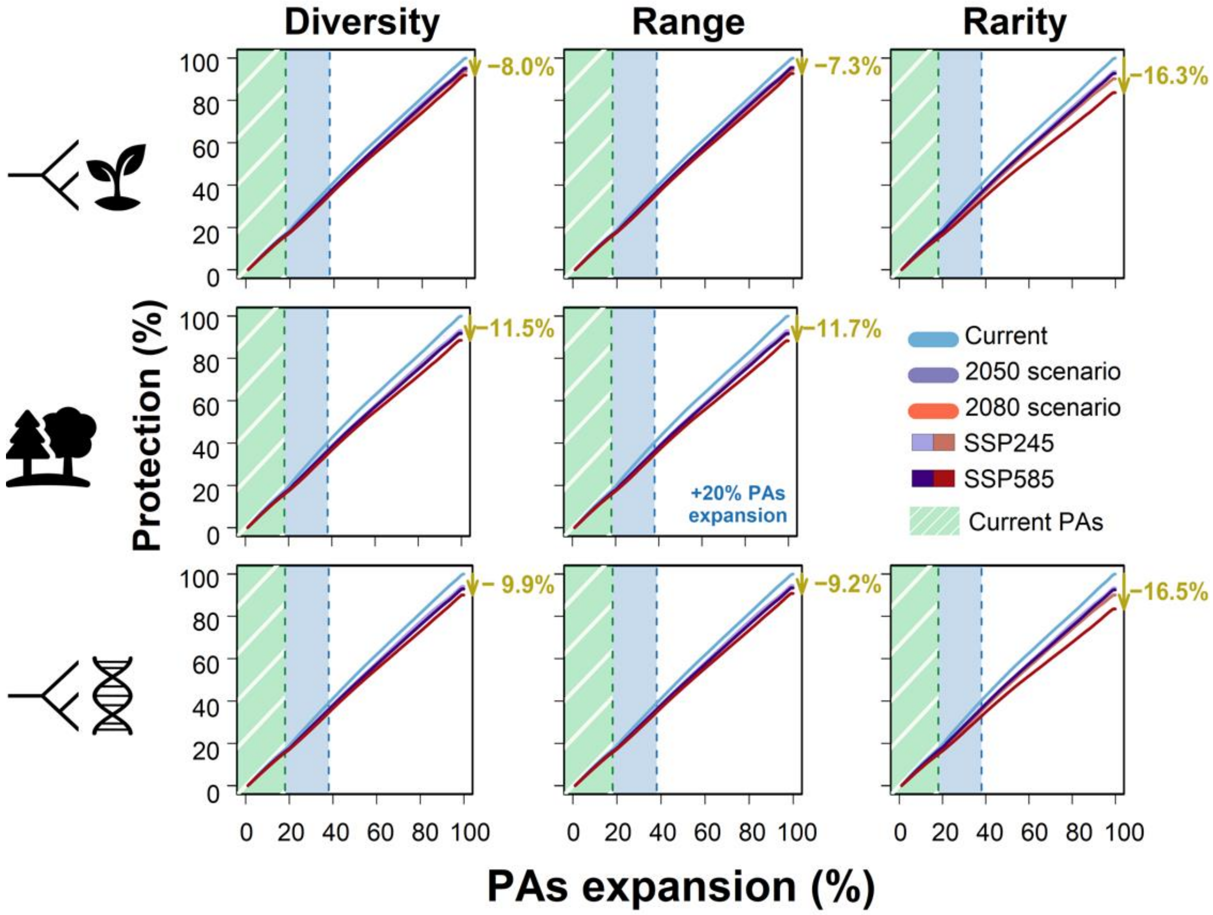

Supplementary Figure 1. Current and future protected areas (PAs) network cumulative expansion in the European Alps when maximizing regional diversity (CAZ algorithm) for limited plant dispersal.

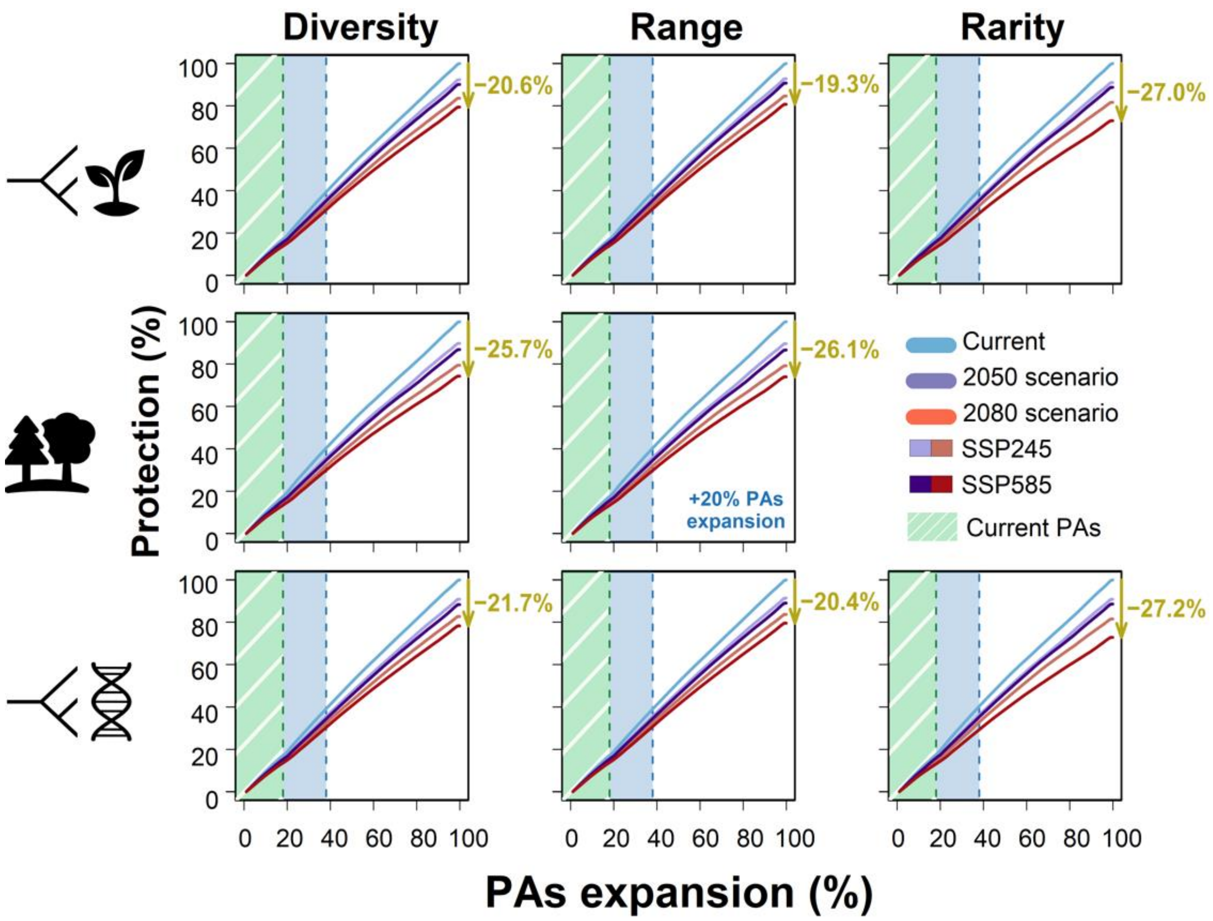

Supplementary Figure 2. Current and future protected areas (PAs) network cumulative expansion in the European Alps when maximizing local diversity (ABF algorithm) for no plant dispersal.

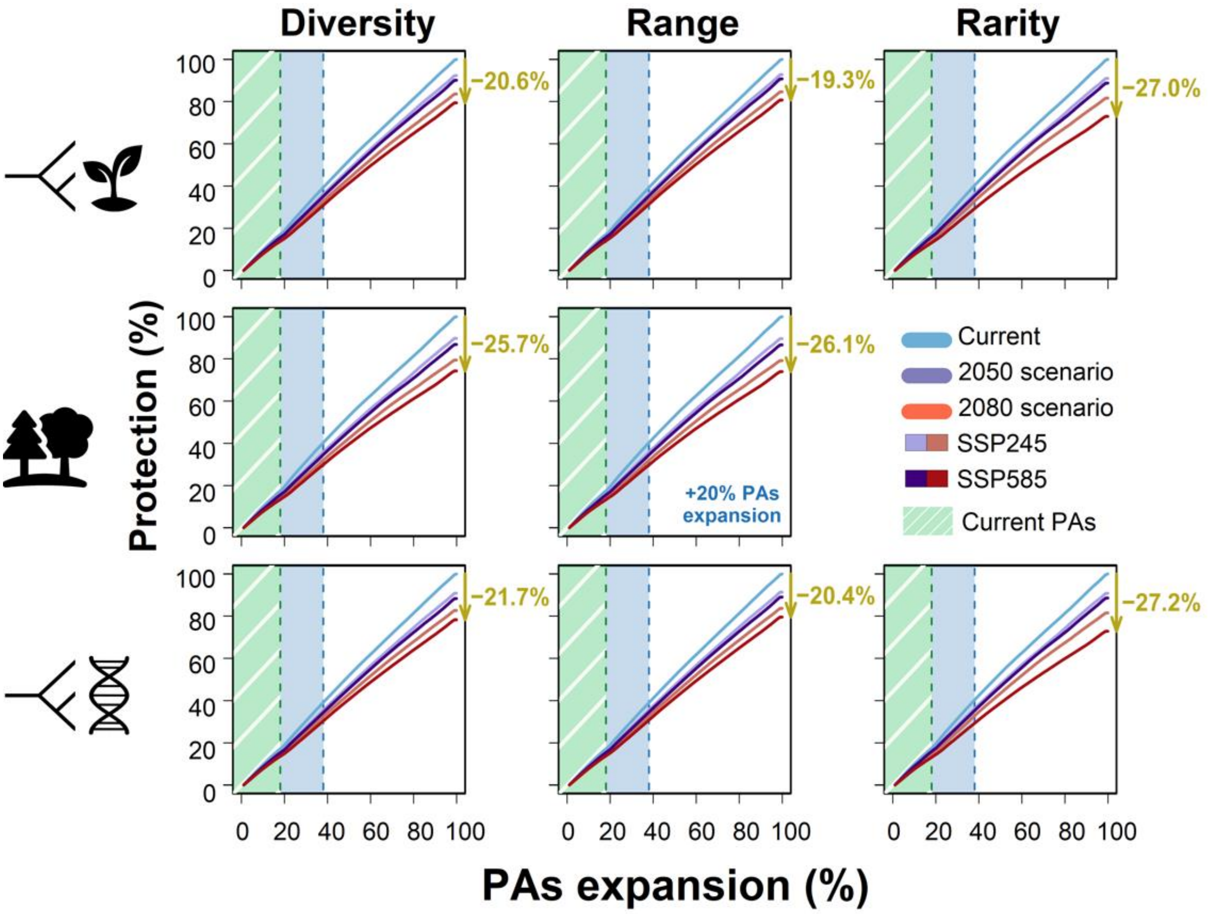

Supplementary Figure 3. Current and future protected areas (PAs) network cumulative expansion in the European Alps when maximizing regional diversity (CAZ algorithm) for no plant dispersal.

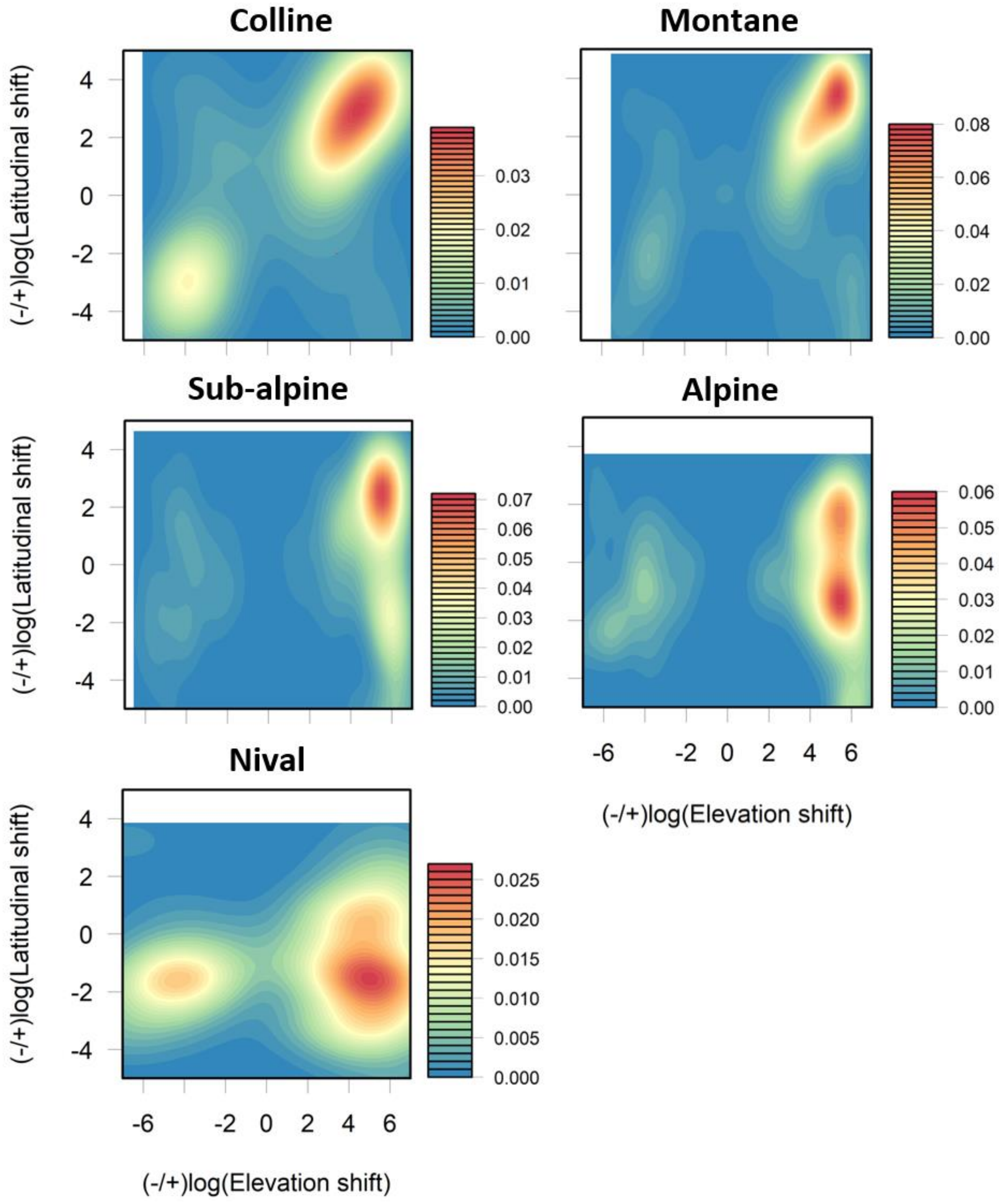

Supplementary Figure 4. Latitudinal and elevation changes in species ranges by 2080 for SSP585 and limited plant dispersal. Heat maps represent, for each species elevation class, the density of its predicted spatial changes in a two-dimensional space.

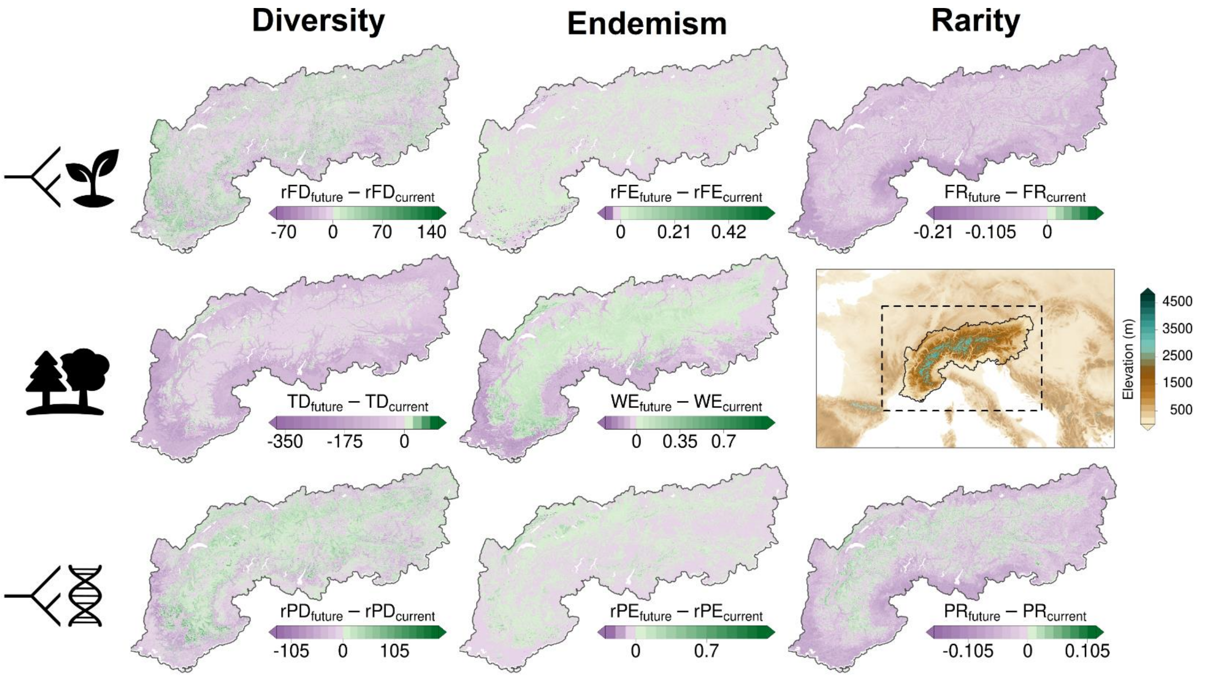

Supplementary Figure 5. Change in multifaceted diversity and uniqueness by 2050 for SSP585 and limited plant dispersal. First, second and third row depict the functional, taxonomic, and phylogenetic dimension respectively. Spatial gains are shown in green, spatial losses are shown in purple. TD, rPD and rFD: taxonomic, relative phylogenetic and relative functional diversity (relative diversity represents the diversity expected under a given taxonomic diversity, see methods); WE, rPE and rFE: weighted taxonomic, relative phylogenetic and functional endemism respectively; PR and FR: phylogenetic and functional rarity.

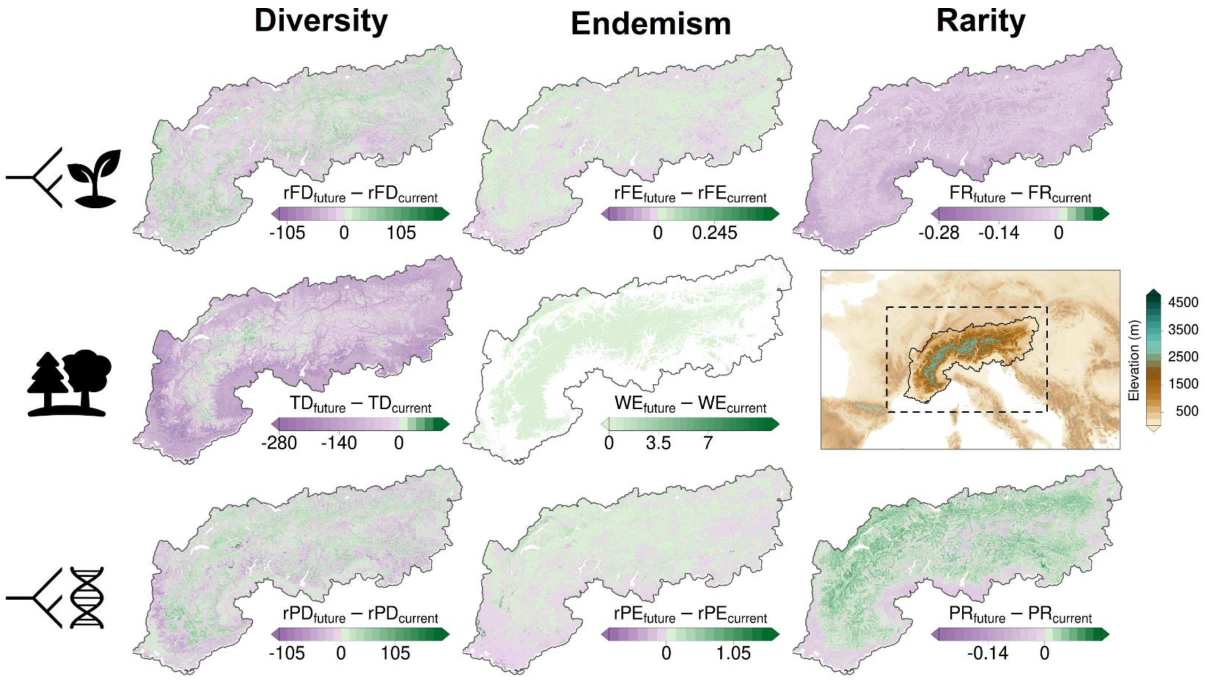

Supplementary Figure 6. Change in multifaceted diversity and uniqueness by 2080 for SSP245 and limited plant dispersal.

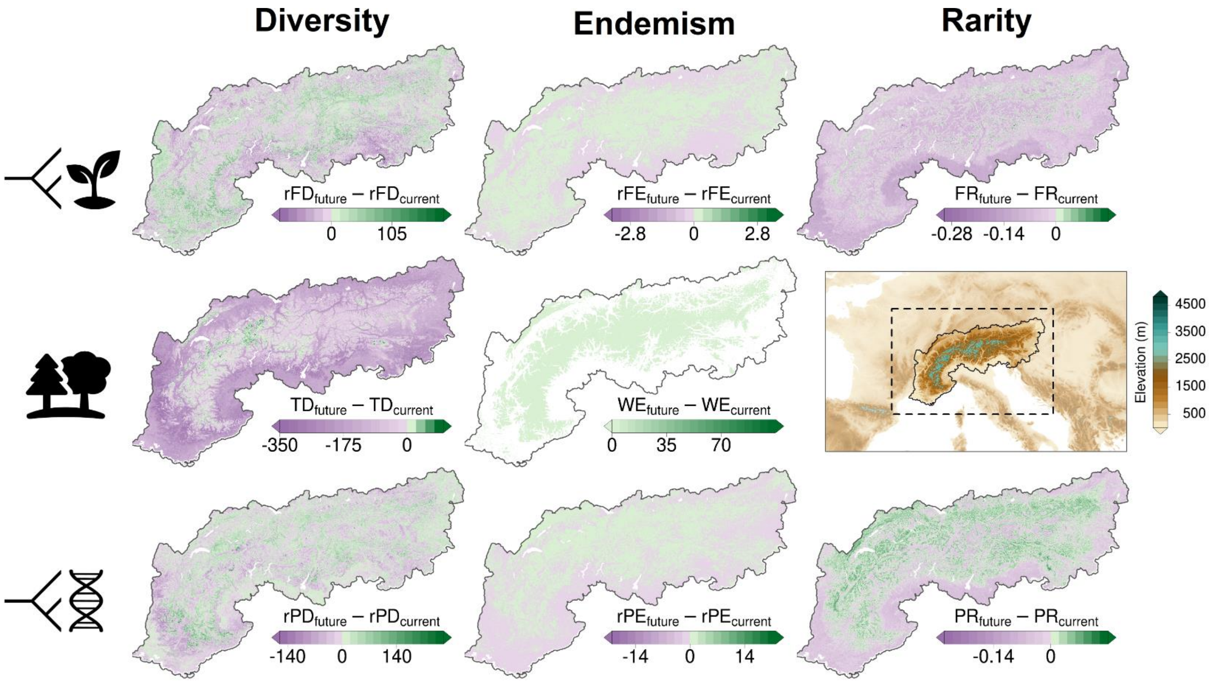

Supplementary Figure 7. Change in multifaceted diversity and uniqueness by 2080 for SSP585 and limited plant dispersal.

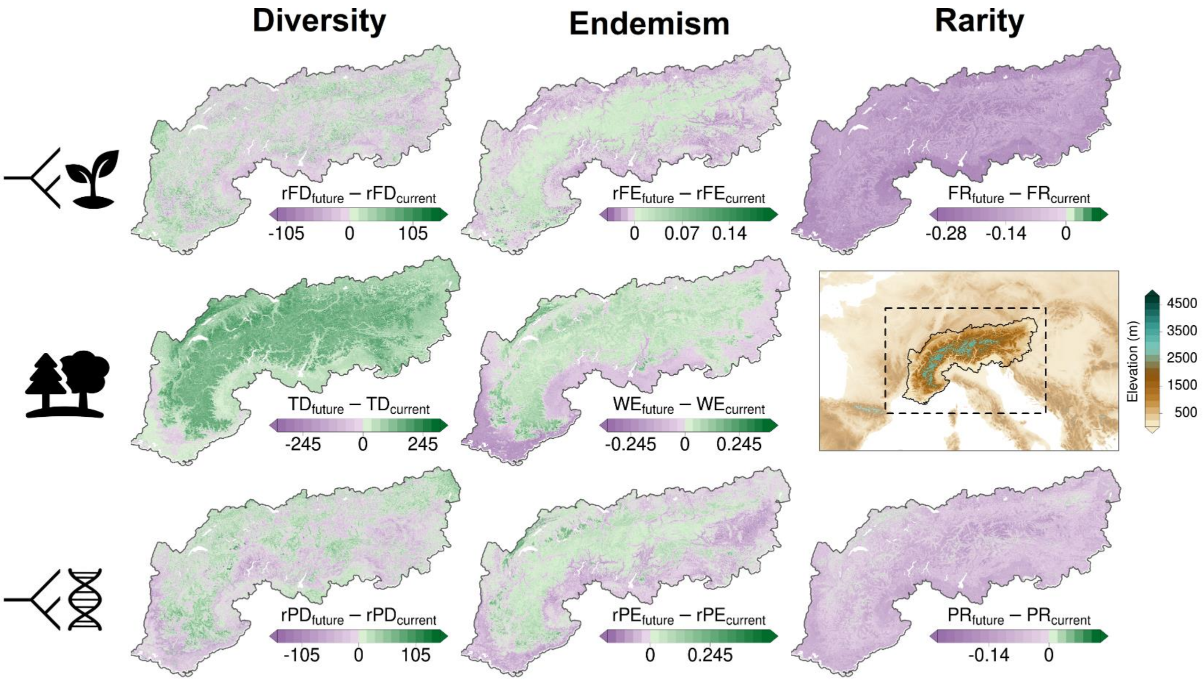

Supplementary Figure 8. Change in multifaceted diversity and uniqueness by 2050 for SSP245 and unlimited plant dispersal.

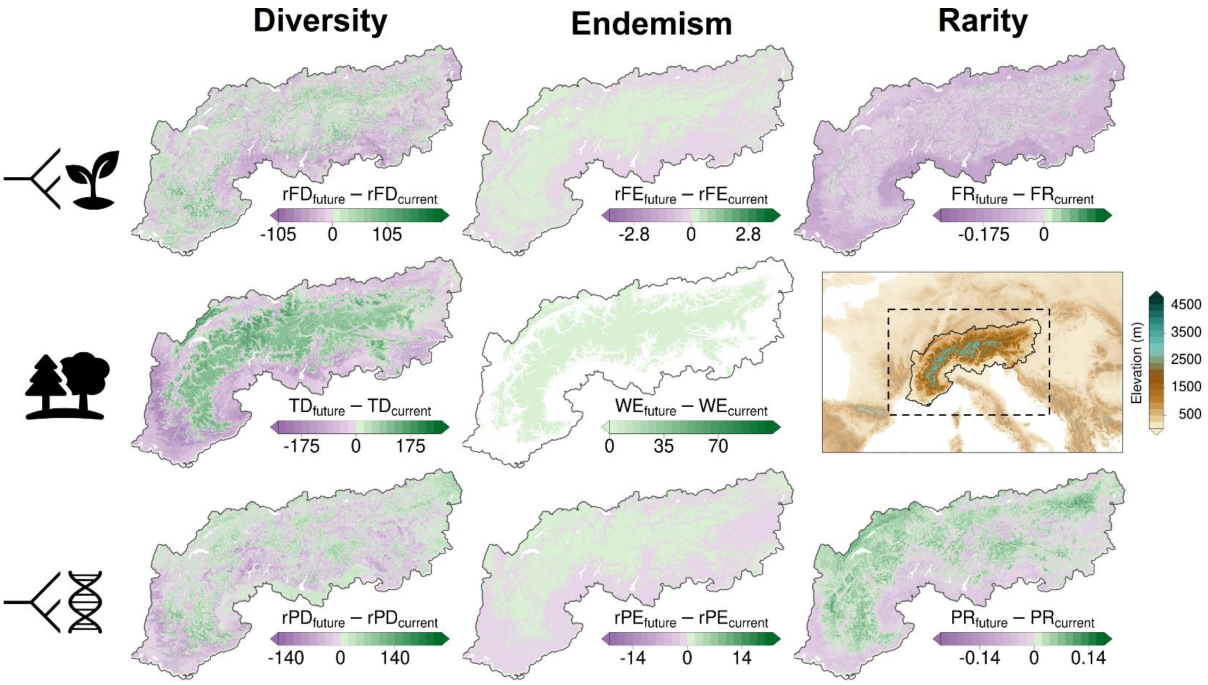

Supplementary Figure 9. Change in multifaceted diversity and uniqueness by 2080 for SSP585 and no plant dispersal.

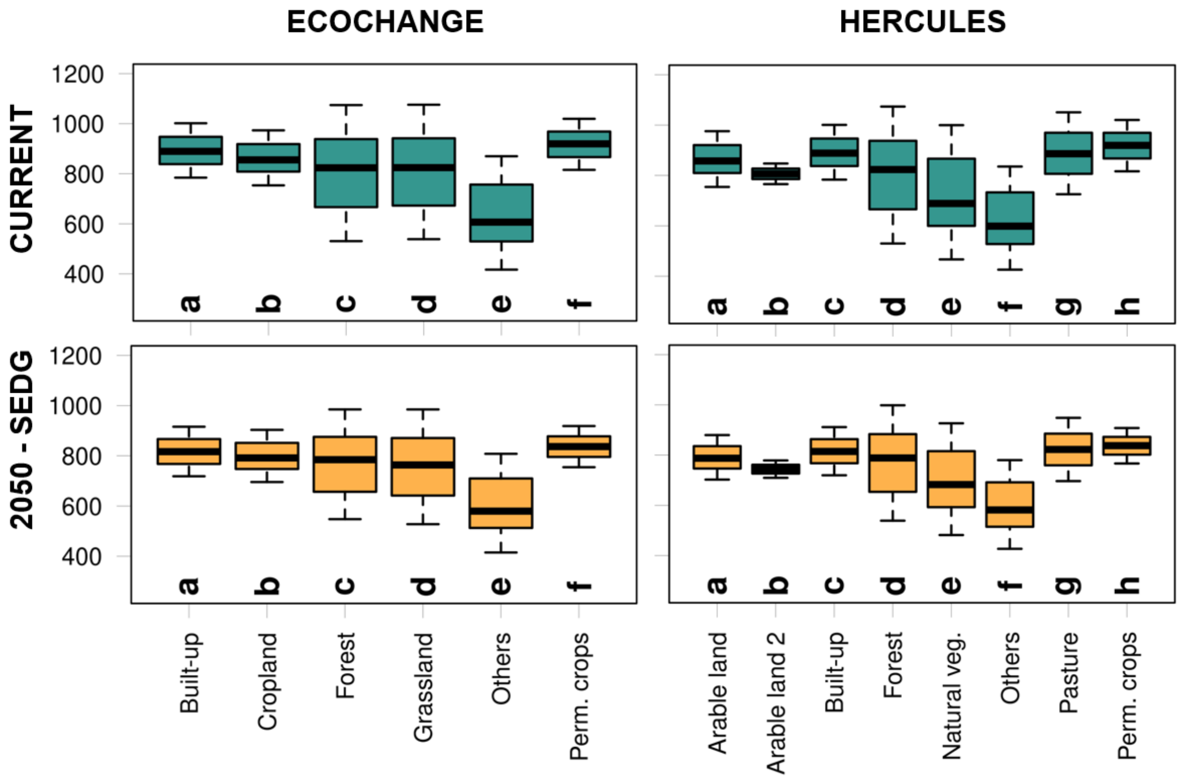

Supplementary Figure 10. Plant species richness distribution by land cover (LC) type for two LC products (ALARME-ECOCHANGE and VOLANTES-HERCULES) and timelines (current and by 2050 for the SEDG/SSP245 scenario). Kruskal-Wallis tests were here applied for each panel (****p*-value ~ 0 for all). All pairwise comparisons (two-sided) were run with post-hoc Dunn tests, Bonferroni correction (adjustment for multiple comparisons) and displayed following a letter-based representation (**p*-value < .05). Left panels: n = 289’474 and 288’981 cells examined over six independent land cover for ‘current’ and ‘2050 – SEDG’ respectively. Right panels: n = 286’104 and 284’868 cells examined over eight independent land cover for ‘current’ and ‘2050 – SEDG’ respectively. Boxplots indicate median (middle line), 25th and 75th percentile (box), and minimum and maximum (whiskers). Perm. crops: Permanent crops. Arable land 2: Irrigated arable land. Natural veg.: Natural vegetation.

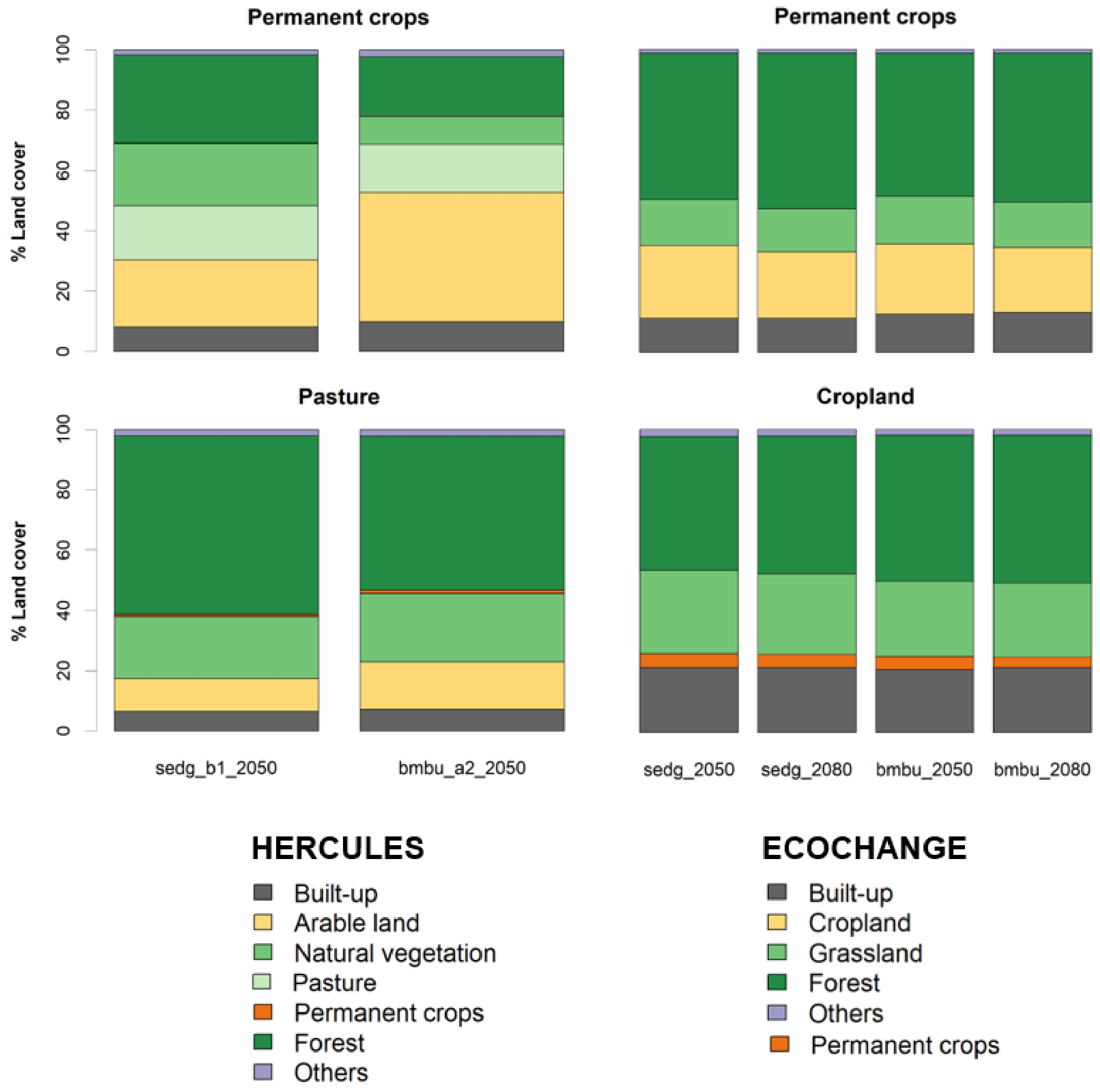

Supplementary Figure 11. Land cover (LC) change (%) from different types of agricultural LC abandonment (Permanent crops, Pasture and Cropland) to other LC classes, for VOLANTES-HERCULES (left panels) and ALARME-ECOCHANGE (right panels), and their associated carbon scenarios (by 2050 and 2080 for SEDG/SSP245 and BMBU/SSP585 scenario). For each future scenario, LC change (%) was calculated as the pixel proportion of new LC types succeeding previous permanent crops, pasture, or cropland. Linked to below, each categorical LC is related to certain level of species richness, therefore, each change also implies a change in diversity.

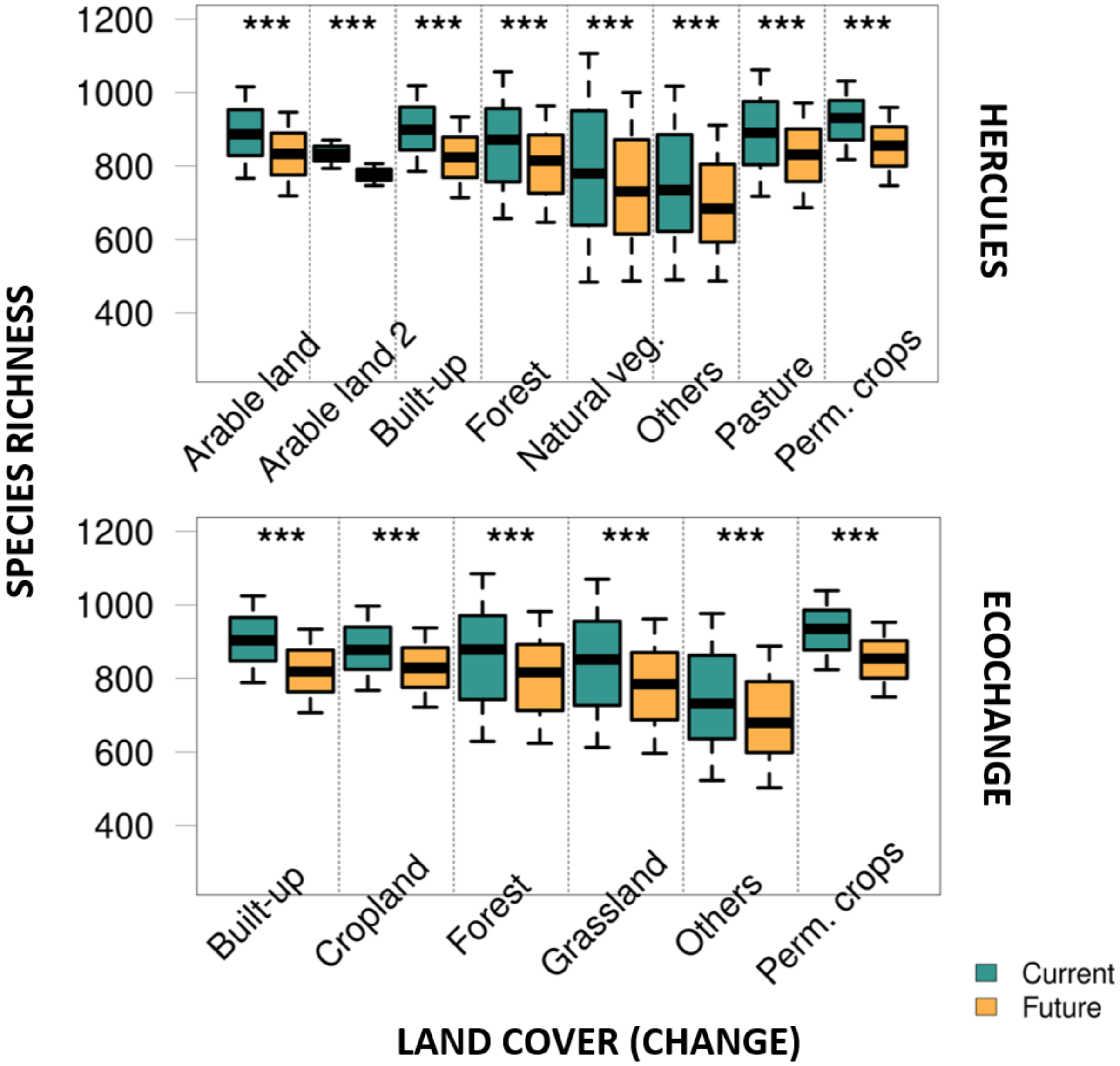

Supplementary Figure 12. Species diversity change according to land cover (LC) change for VOLANTES-HERCULES (top panel) and ALARME-ECOCHANGE (bottom panel), by 2050 and for the SEDG/SSP245 scenario. Unlike Supplementary Figure 10, diversity distributions were not extracted from the whole the study region, but only from pixels enduring a change in a specific LC type (labels), i.e., same pixels for both original (current) and transformed LC (future). Each current and future diversity distribution were statistically compared with Wilcoxon unpaired two-sided tests (****p*-value ~ 0 for all comparisons). Top panel: n = 201'224 cells examined over eight independent land cover and two different timelines. Bottom panel: n = 144'750 cells examined over six independent land cover and two different timelines. Boxplots indicate median (middle line), 25th and 75th percentile (box), and minimum and maximum (whiskers). Perm. crops: Permanent crops. Arable land 2: Irrigated arable land. Natural veg.: Natural vegetation.

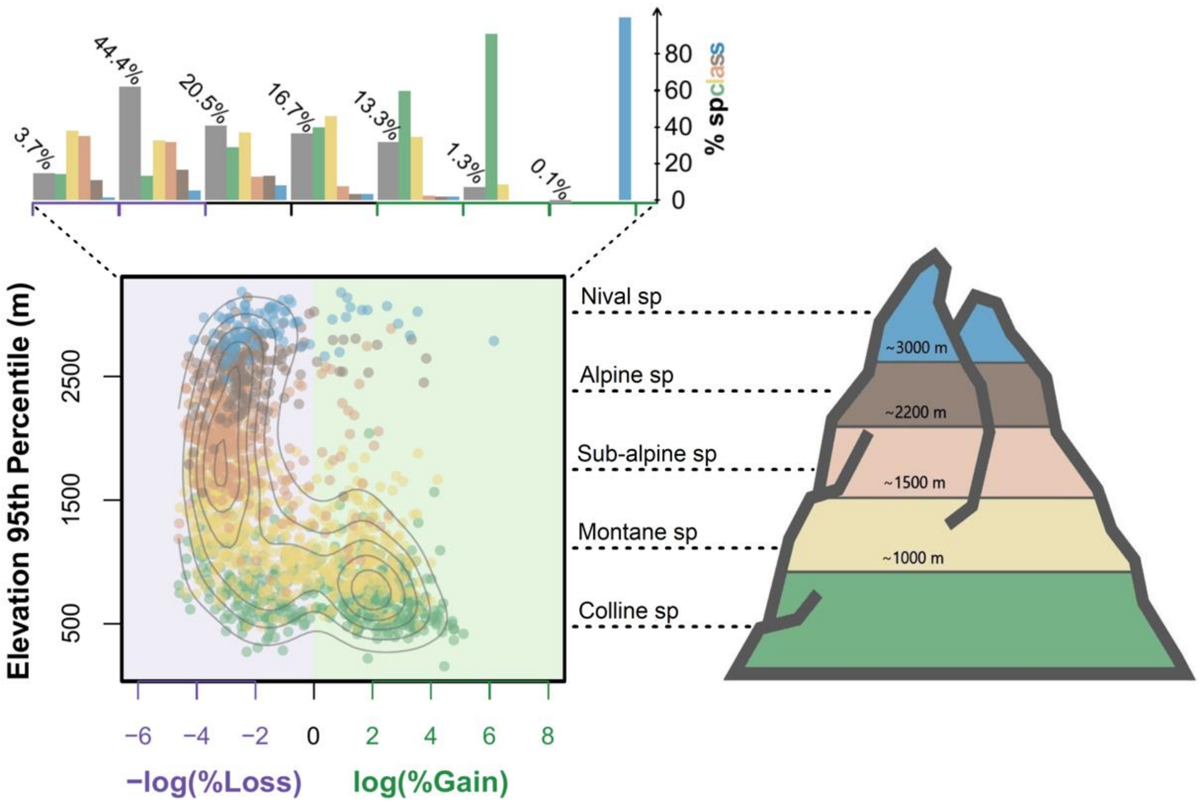

Supplementary Figure 13. Species range shifts by 2050 for SSP585 and limited plant dispersal.

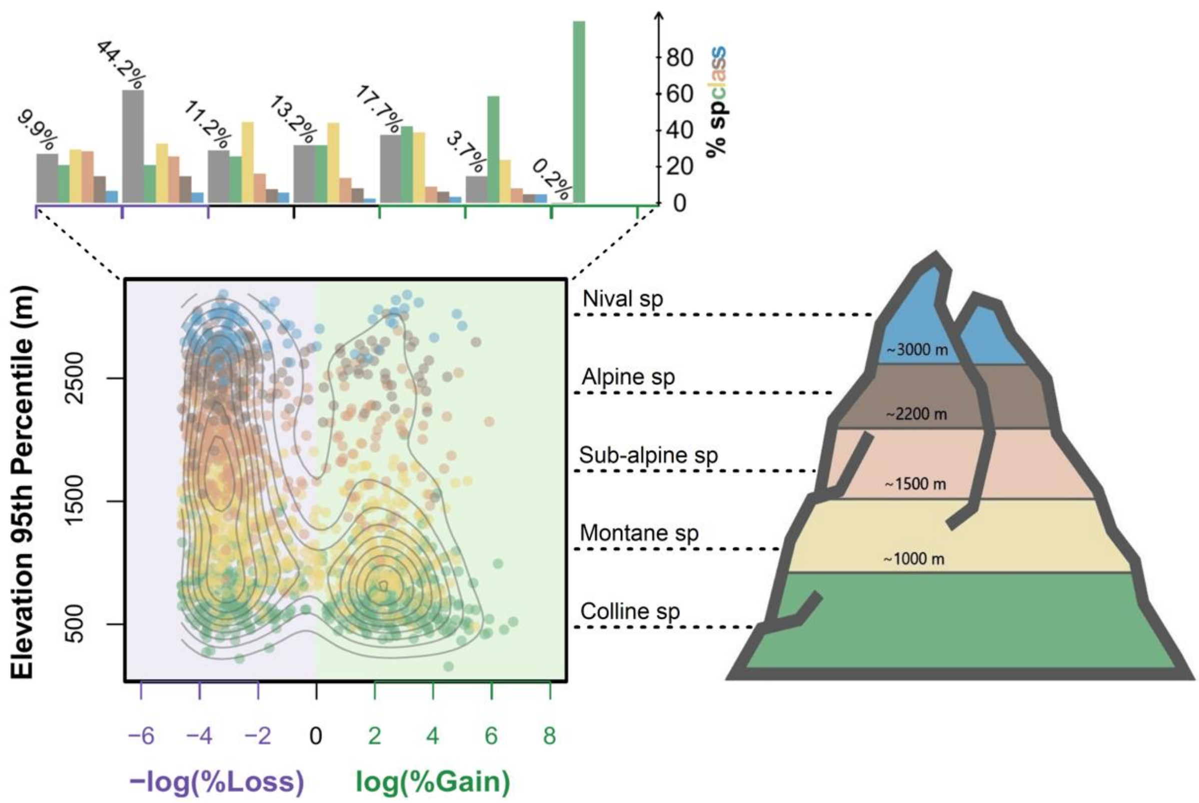

Supplementary Figure 14. Species range shifts by 2080 for SSP245 and limited plant dispersal.

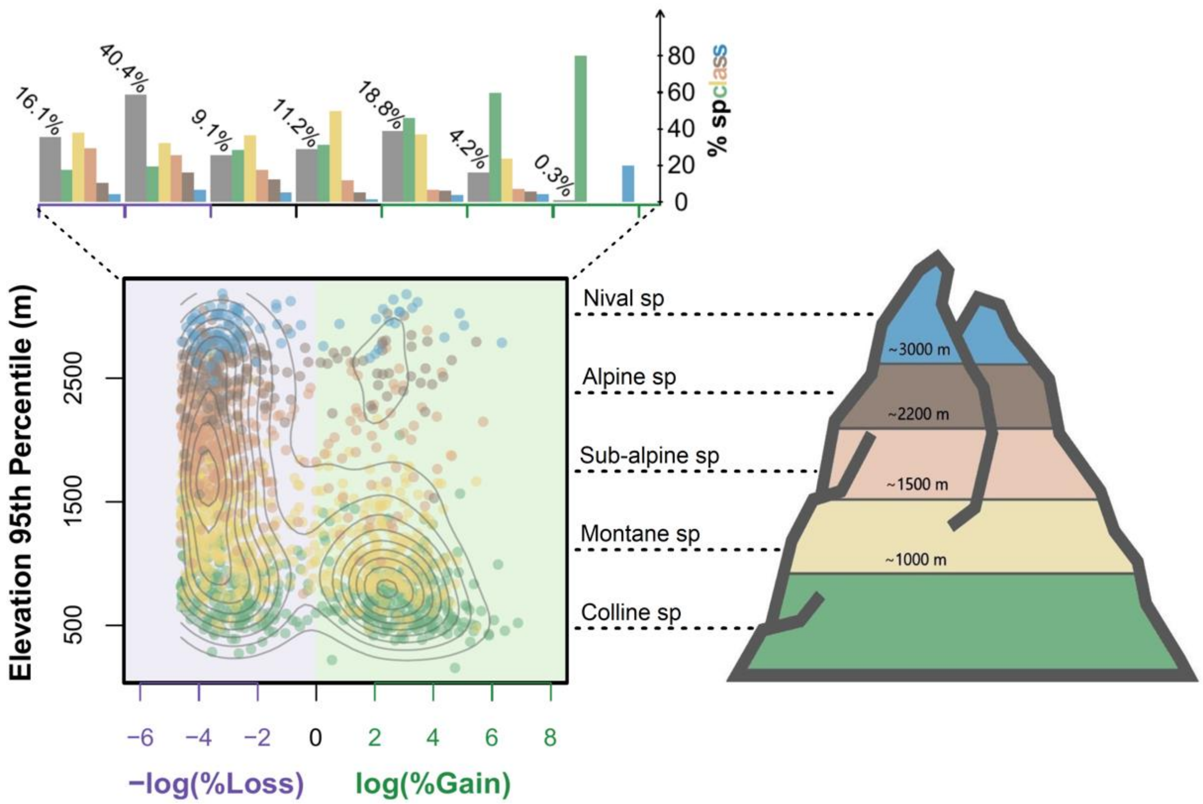

Supplementary Figure 15. Species range shifts by 2080 for SSP585 and limited plant dispersal.

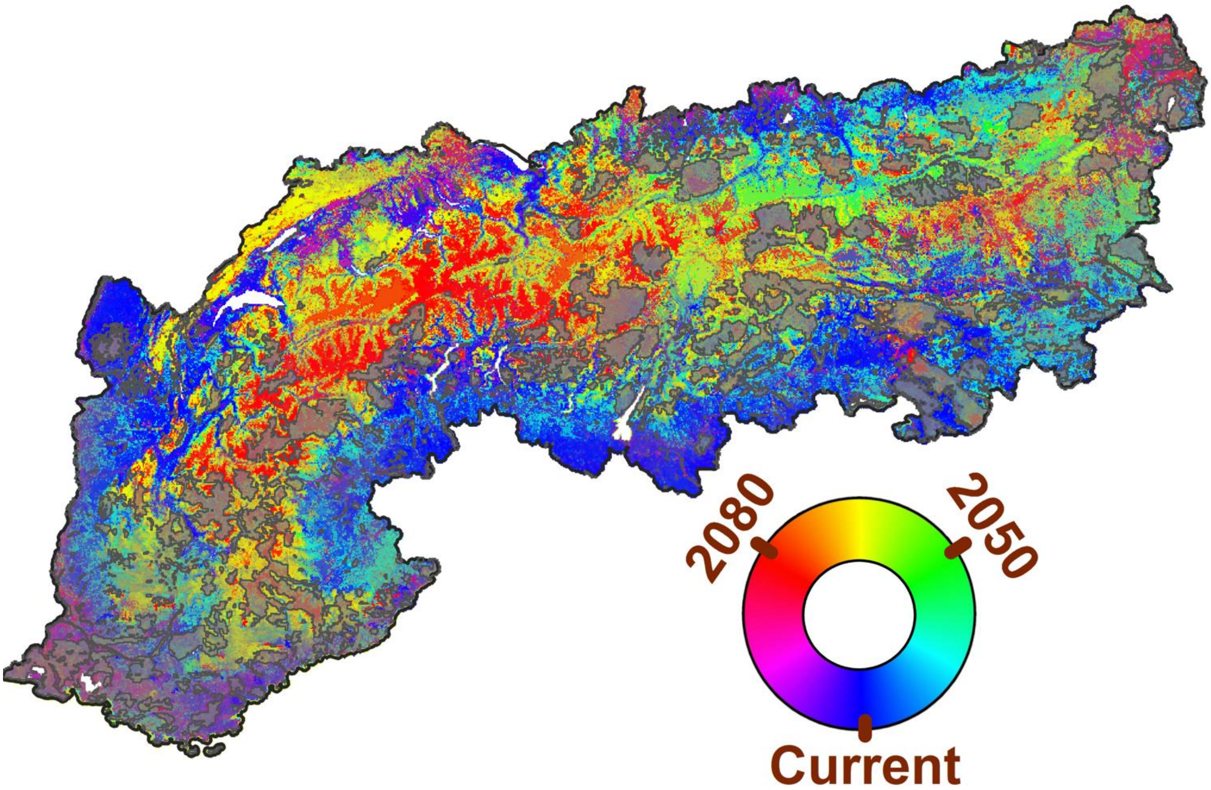

Supplementary Figure 16. Current and future local conservation hotspots in the European Alps for SSP585 and limited plant dispersal, under *Zonation* ABF simulations of reserve network expansion (‘expansion’ approach). Grey with contour lines describes the current IUCN I-II and Natura 2000 PA network.

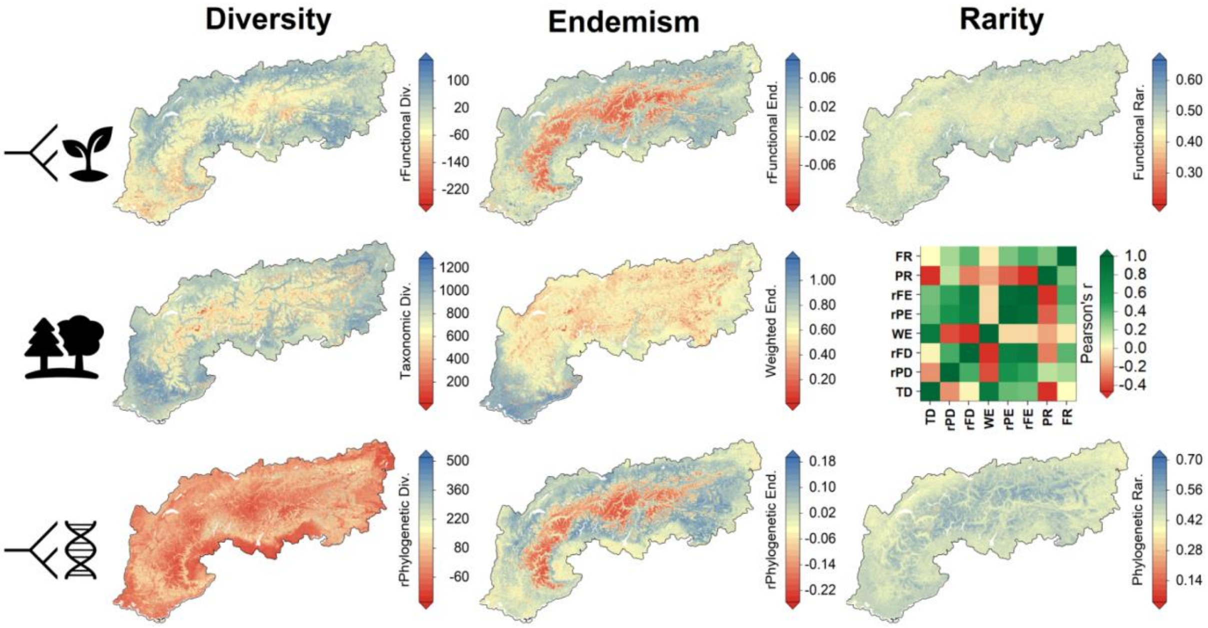

Supplementary Figure 17. Current distribution of multifaceted diversity and uniqueness in the European Alps. First, second and third row depict the functional, taxonomic and phylogenetic dimension respectively. Middle right raster plot shows Pearson's correlation relationships between all maps. TD, rPD and rFD: taxonomic, relative phylogenetic and functional diversity; WE, rPE and rFE: weighted taxonomic, relative phylogenetic and functional endemism respectively; PR and FR: phylogenetic and functional rarity.

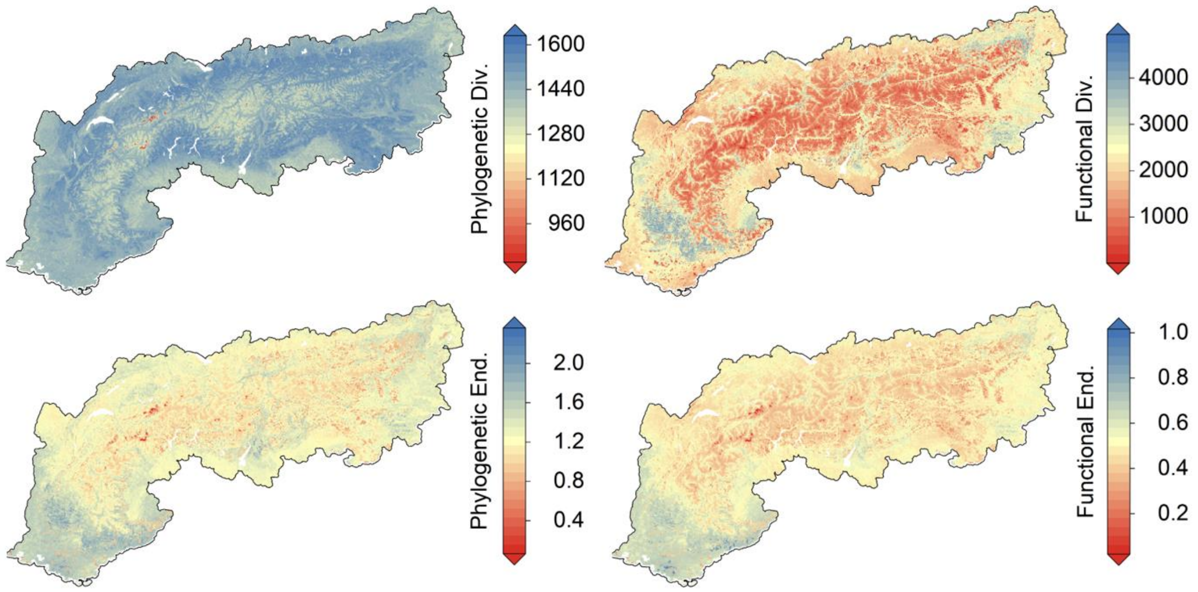

Supplementary Figure 18. Distribution of current phylogenetic, functional diversity and endemism in the European Alps. Here are depicted the absolute version of the four diversity features.

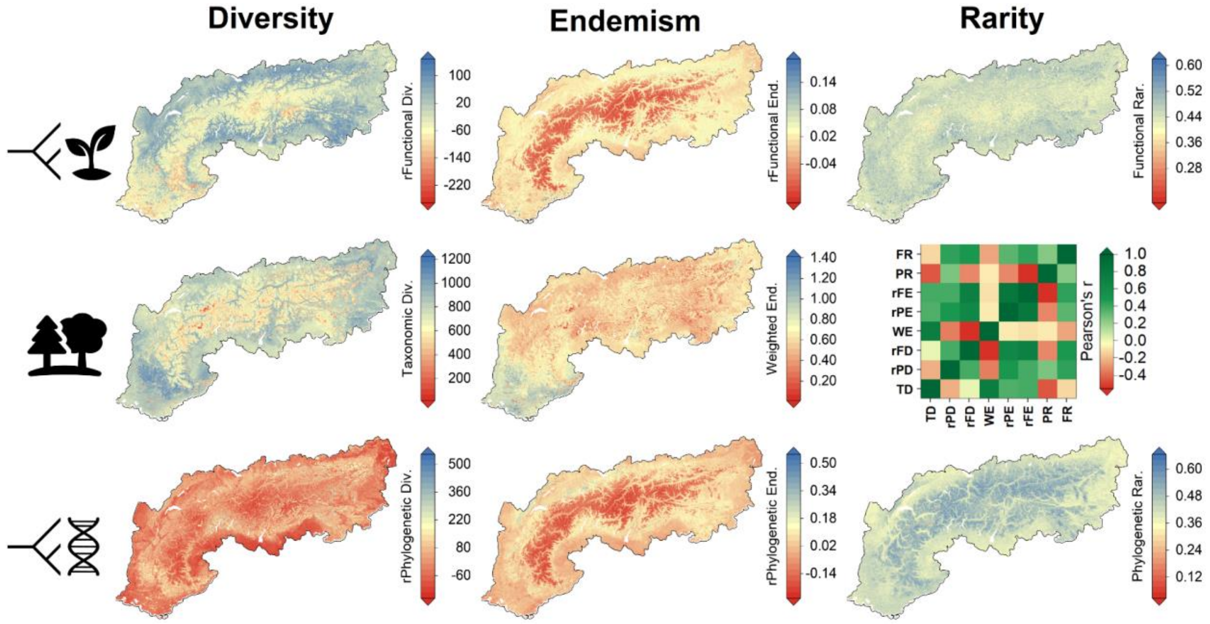

Supplementary Figure 19. Distribution of multifaceted diversity and uniqueness in the European Alps by 2050 for SSP245 and limited plant dispersal.

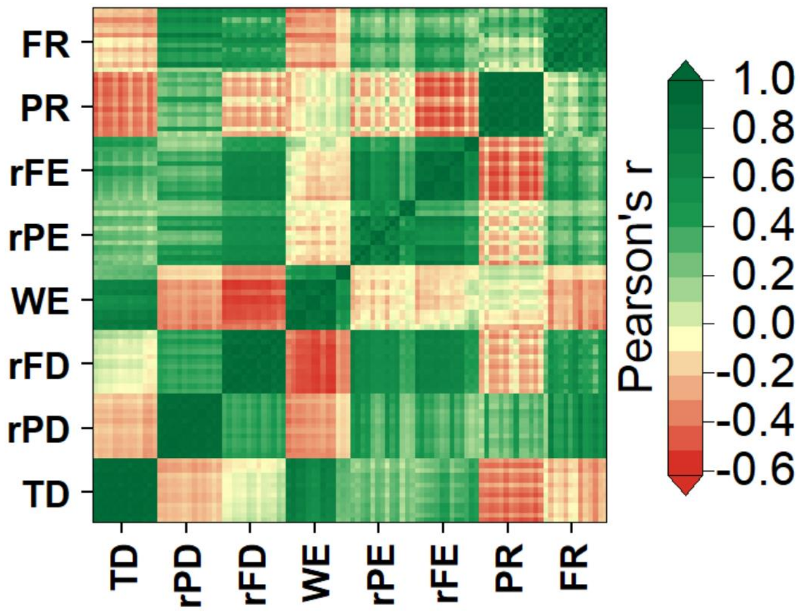

Supplementary Figure 20. Pearson's correlation relationships between all multifaceted diversity and uniqueness maps generated for the study. Correlations are assessed for a total of 104 diversity and uniqueness maps. Each x and y axis label includes in total 13 maps, i.e., per current and 12 future timeline/SSP/dispersal scenarios. Overall, general patterns follow the correlation relationships found above. TD, rPD and rFD: taxonomic, relative phylogenetic and functional diversity; WE, rPE and rFE: weighted taxonomic, relative phylogenetic and functional endemism respectively; PR and FR: phylogenetic and functional rarity.

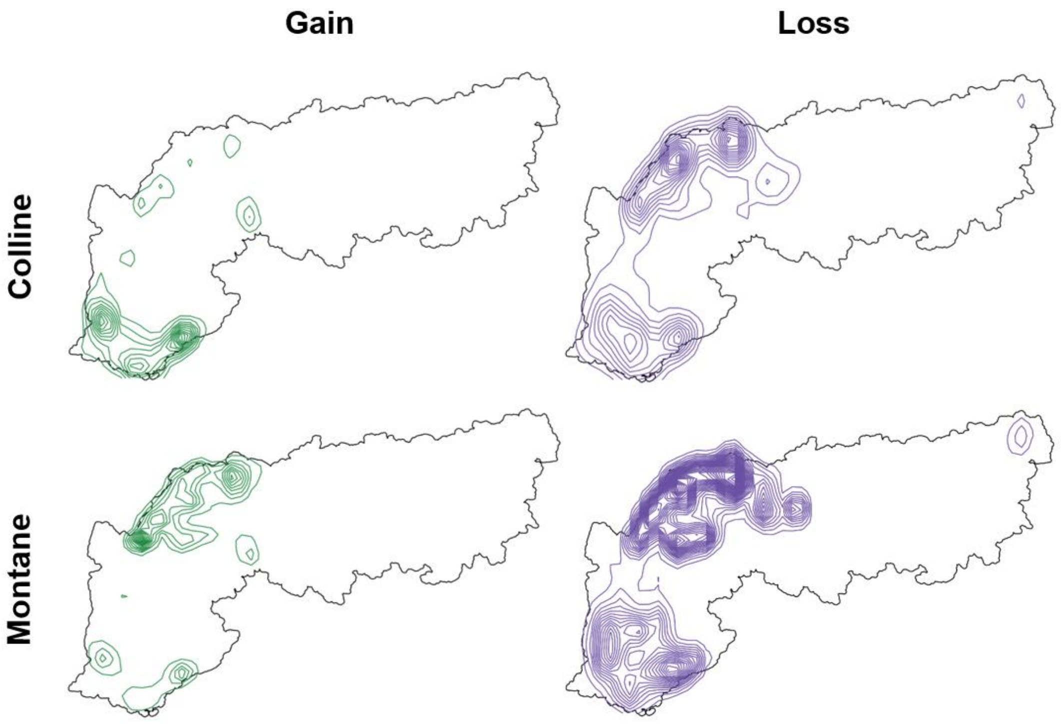

Supplementary Figure 21. Observation density of colline (upper) and montane species (lower) undergoing high future range gain (left) and loss (right) by 2050 for SSP245 and limited plant dispersal. High gains and losses are defined as > 75th quantile of all species' range gains and losses, respectively. Green and purple upper contour lines summarize the observation density of 65 (n = 82,133 records) and 56 species (n = 29,762 records) respectively. Green and purple lower contour lines summarize the density of 61 (n = 144,776 records) and 94 species (n = 72,642 records) respectively.

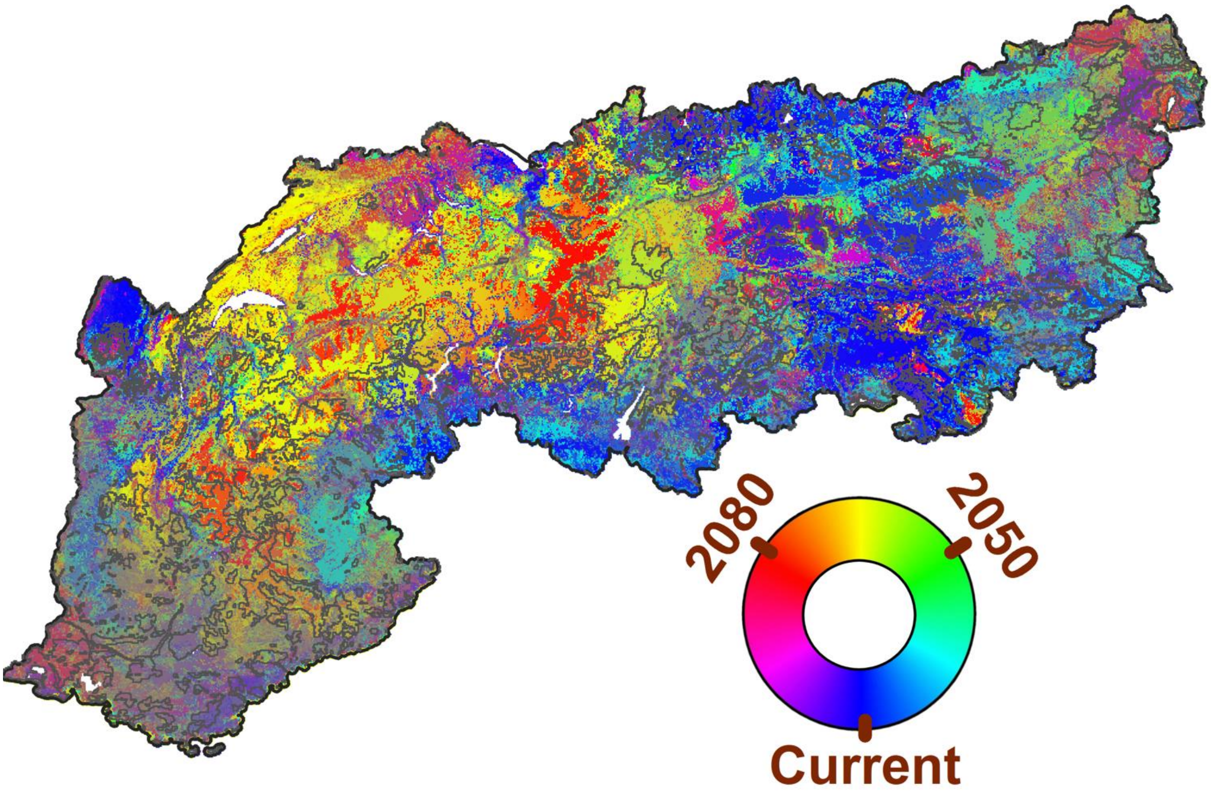

Supplementary Figure 22. Current and future local conservation hotspots in the European Alps for SSP245 and limited plant dispersal, under *Zonation* ABF simulations of optimal reserve selection (‘selection’ approach). Contour lines describes the current IUCN I-II and Natura 2000 PA network.

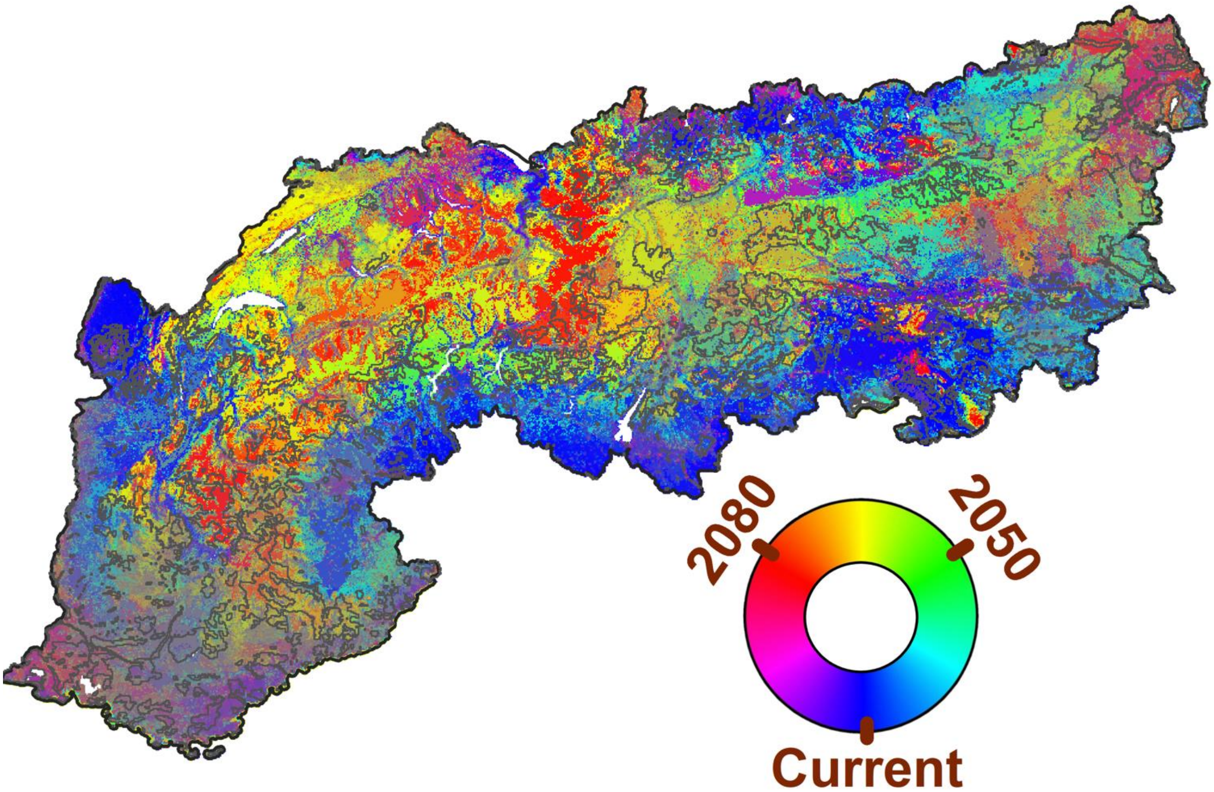

Supplementary Figure 23. Current and future local conservation hotspots in the European Alps for SSP585 and limited plant dispersal, under *Zonation* ABF simulations of optimal reserve selection (‘selection’ approach). Contour lines describes the current IUCN I-II and Natura 2000 PA network.

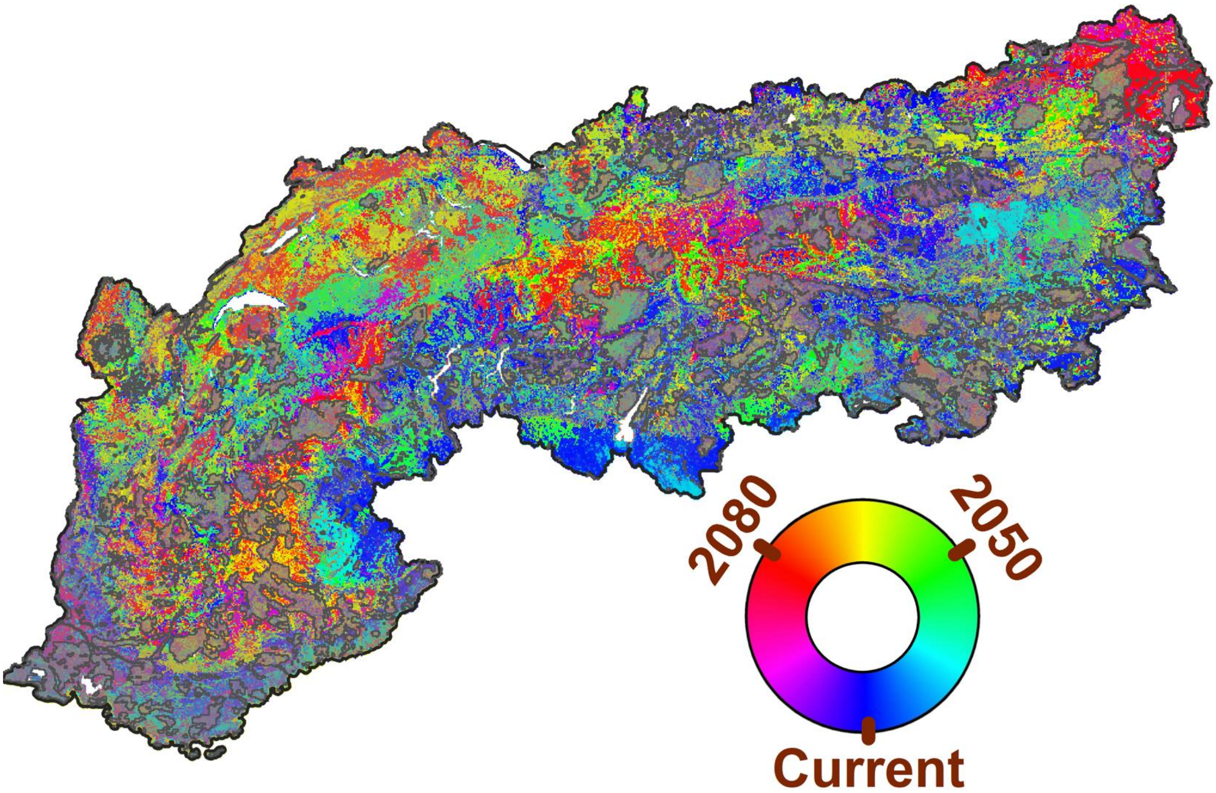

Supplementary Figure 24. Current and future regional conservation hotspots in the European Alps for SSP245 and limited plant dispersal, under *Zonation* CAZ simulations of reserve network expansion (‘expansion’ approach). Grey with contour lines describes the current IUCN I-II and Natura 2000 PA network.

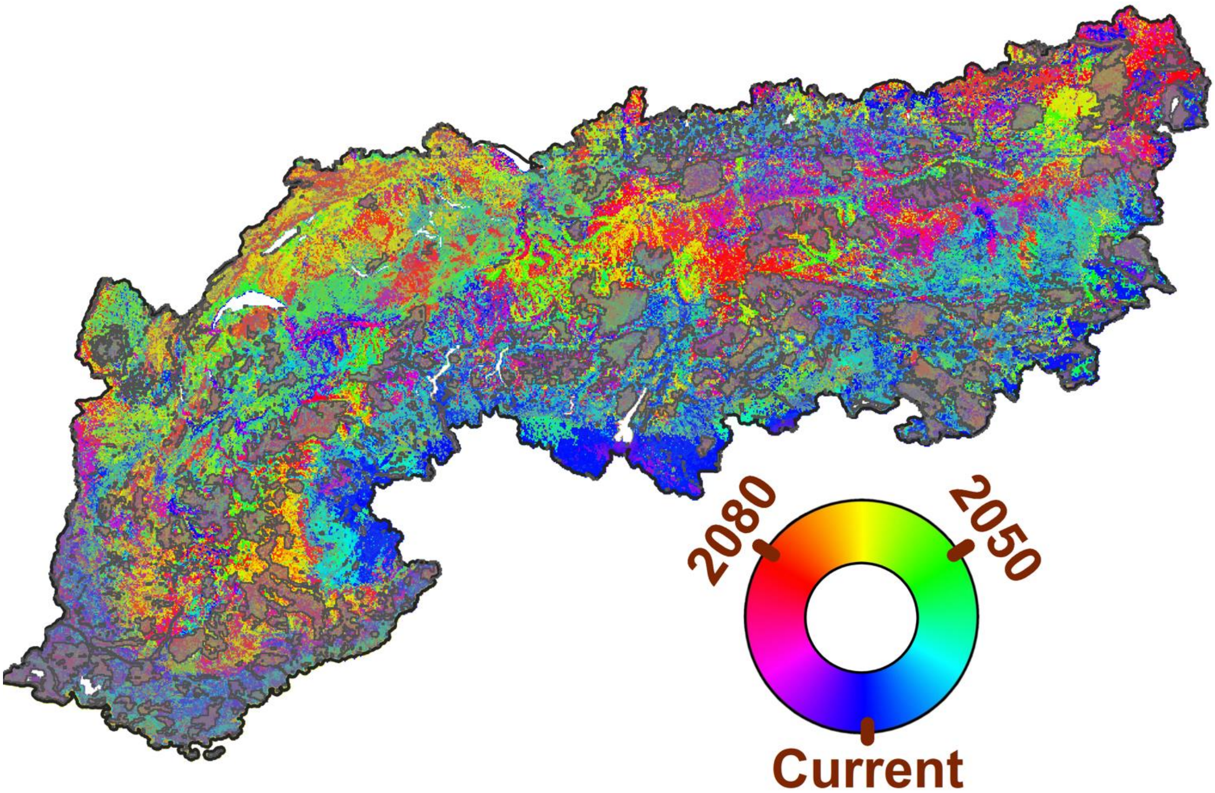

Supplementary Figure 25. Current and future regional conservation hotspots in the European Alps for SSP585 and limited plant dispersal, under *Zonation* CAZ simulations of reserve network expansion (‘expansion’ approach). Grey with contour lines describes the current IUCN I-II and Natura 2000 PA network.

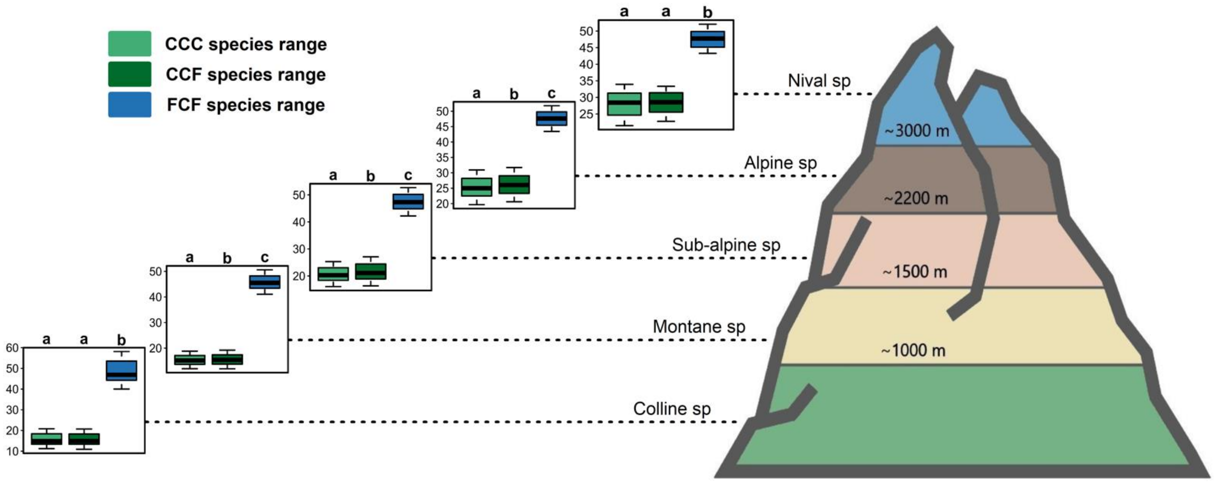

Supplementary Figure 26. Summary of species range protection (%) for current (green) and expanded PAs (blue; *Zonation* CAZ algorithm) in 2050 under SSP245 and limited plant dispersal. Expanded PAs represents the top 20% regional expansion of the reserve network of the European Alps. CCC: current conservation of current species range, CCF: current conservation of future species range, FCF: future conservation of future species range. *Friedman tests* were applied here for each panel (****p*-value ~ 0 for all) to compare the median values of the three boxplots. All pairwise comparisons (two-sided) were run with post-hoc *Nemenyi tests* and displayed following a letter-based representation (**p-value* < 0.05). Top to bottom panel: n = 84, 192, 336, 616 and 483 species respectively examined over three independent treatments. Boxplots indicate median (middle line), 25th and 75th percentile (box), and minimum and maximum (whiskers).

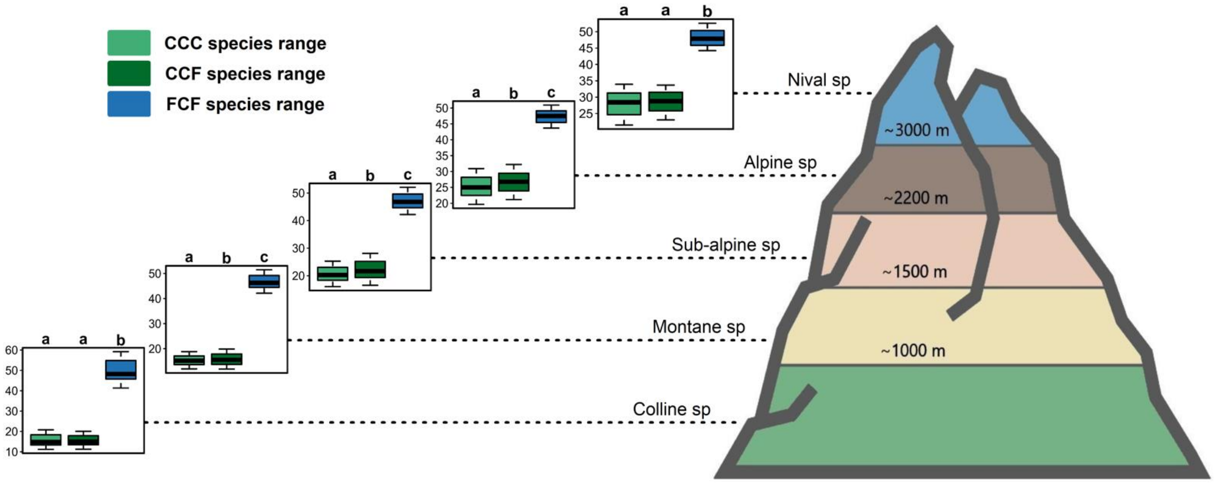

Supplementary Figure 27. Summary of species range protection for current (green) and expanded PAs (blue; *Zonation* CAZ algorithm) in 2050 under SSP585 and limited plant dispersal. Expanded PAs represents the top 20% regional expansion of the reserve network of the European Alps. CCC: current conservation of current species range, CCF: current conservation of future species range, FCF: future conservation of future species range. *Friedman tests* were applied here for each panel (****p*-value ~ 0 for all) to compare the median values of the three boxplots. All pairwise comparisons (two-sided) were run with post-hoc *Nemenyi tests* and displayed following a letter-based representation (**p-value* < 0.05). Top to bottom panel: n = 84, 192, 336, 616 and 483 species respectively examined over three independent treatments. Boxplots indicate median (middle line), 25th and 75th percentile (box), and minimum and maximum (whiskers).

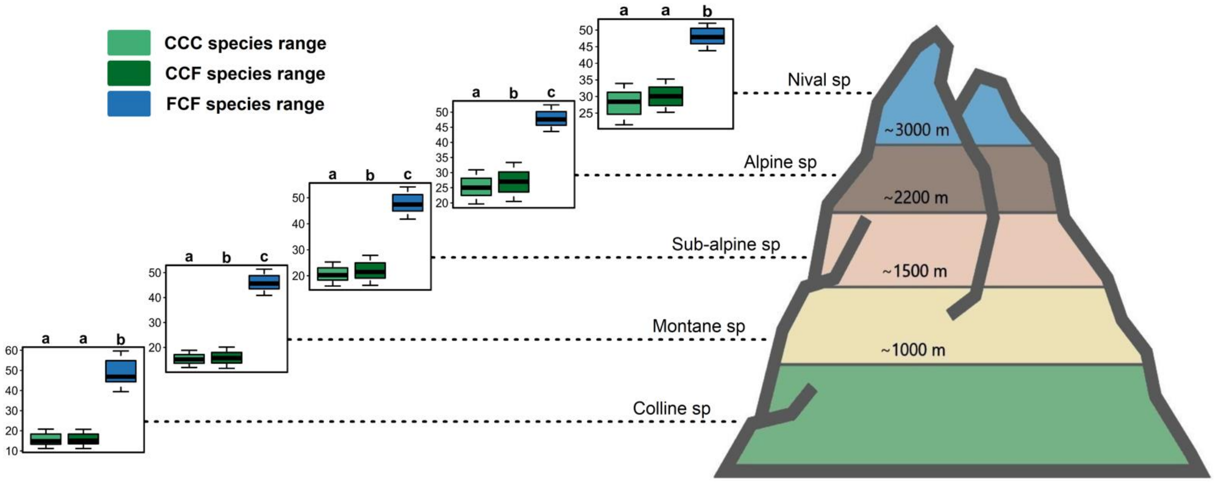

Supplementary Figure 28. Summary of species range protection for current (green) and expanded PAs (blue; *Zonation* CAZ algorithm) in 2080 under SSP245 and limited plant dispersal. Expanded PAs represents the top 20% regional expansion of the reserve network of the European Alps. CCC: current conservation of current species range, CCF: current conservation of future species range, FCF: future conservation of future species range. *Friedman tests* were applied here for each panel (****p*-value ~ 0 for all) to compare the median values of the three boxplots. All pairwise comparisons (two-sided) were run with post-hoc *Nemenyi tests* and displayed following a letter-based representation (**p-value* < 0.05). Top to bottom panel: n = 84, 192, 336, 616 and 483 species respectively examined over three independent treatments. Boxplots indicate median (middle line), 25th and 75th percentile (box), and minimum and maximum (whiskers).

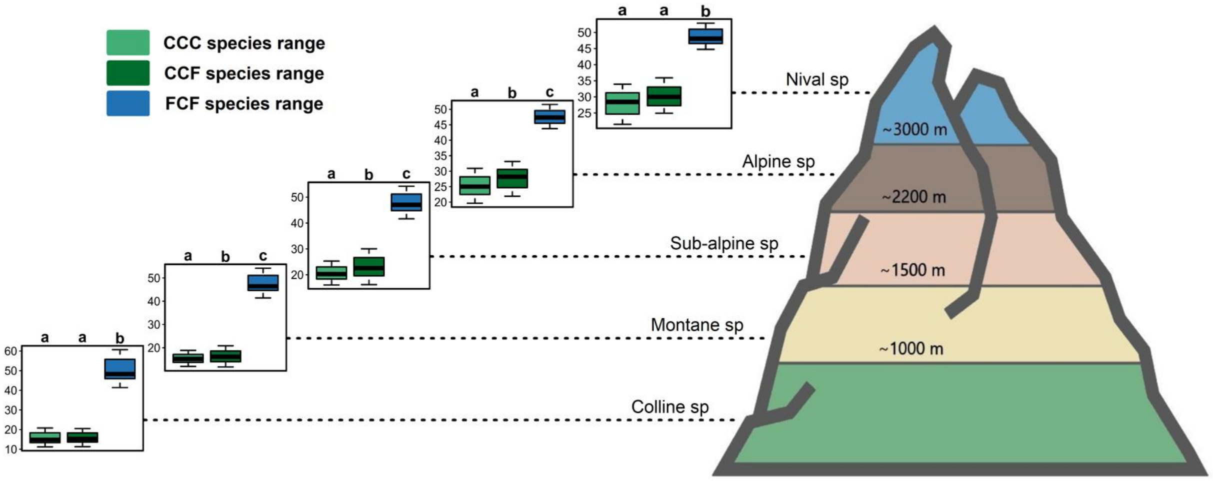

Supplementary Figure 29. Summary of species range protection for current (green) and expanded PAs (blue; *Zonation* CAZ algorithm) in 2080 under SSP585 and limited plant dispersal. Expanded PAs represents the top 20% regional expansion of the reserve network of the European Alps. CCC: current conservation of current species range, CCF: current conservation of future species range, FCF: future conservation of future species range. *Friedman tests* were applied here for each panel (****p*-value ~ 0 for all) to compare the median values of the three boxplots. All pairwise comparisons (two-sided) were run with post-hoc *Nemenyi tests* and displayed following a letter-based representation (**p-value* < 0.05). Top to bottom panel: n = 84, 192, 336, 616 and 483 species respectively examined over three independent treatments. Boxplots indicate median (middle line), 25th and 75th percentile (box), and minimum and maximum (whiskers).

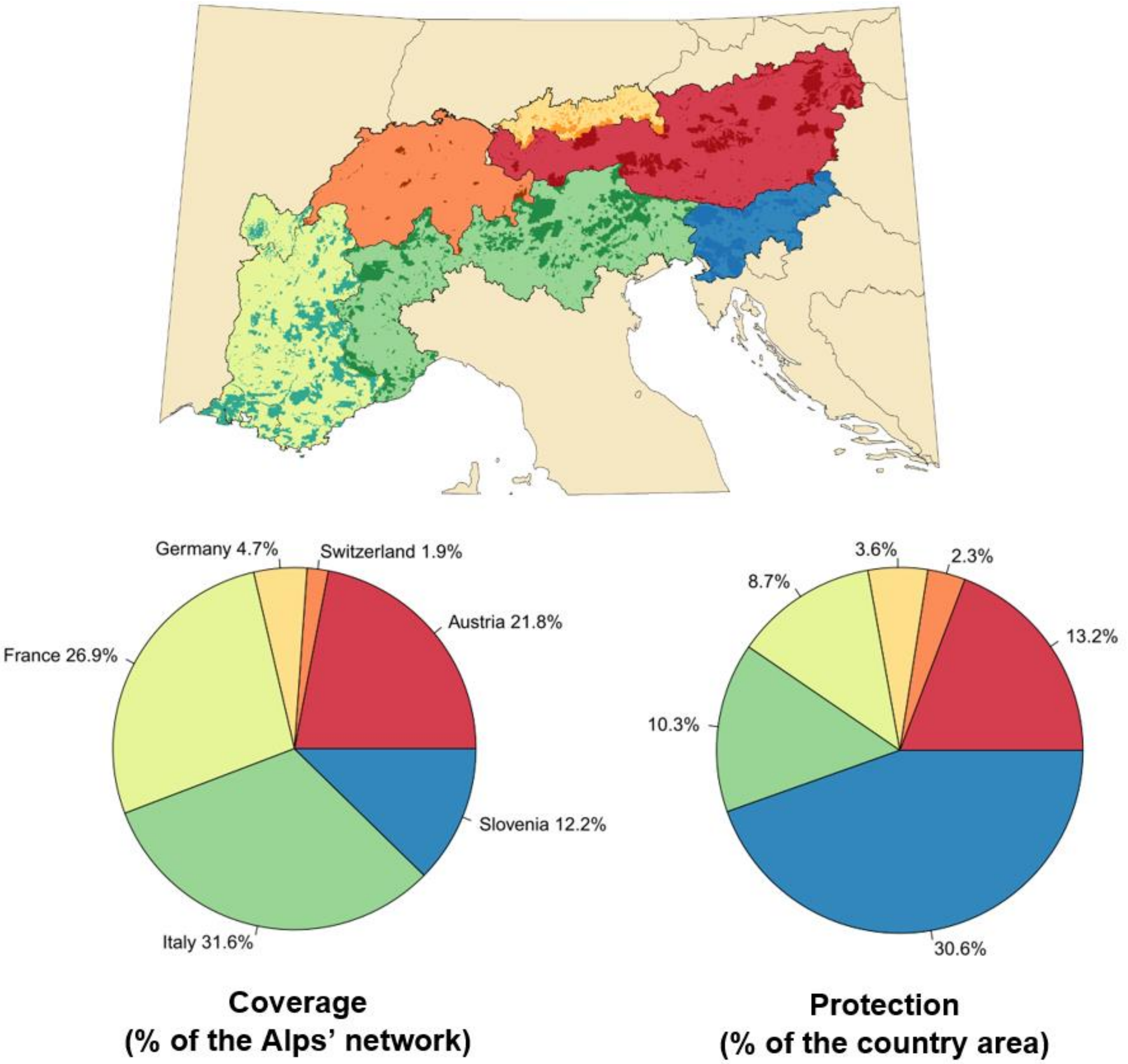

Supplementary Figure 30. Spatial coverage and protection statistics of the European Alps' reserve network (IUCN categories I-II and Natura 2000) for the six main countries member of the Alpine Convention. Left panel depicts the area of the reserve network (in %) covered by each country. Right panel depicts the percentage of area protection relative to the countries' area.

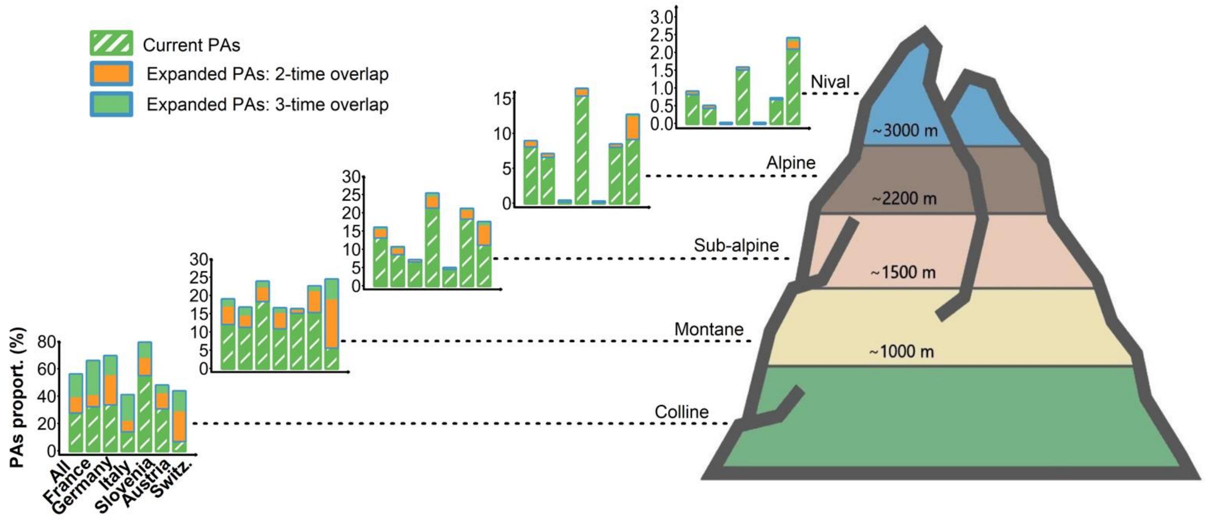

Supplementary Figure 31. Distribution of the current protected areas (PAs) network of the European Alps and its future (top 20%) regional expansion (*Zonation* CAZ algorithm) for SSP245, limited plant dispersal and considering IUCN I-VI categories.

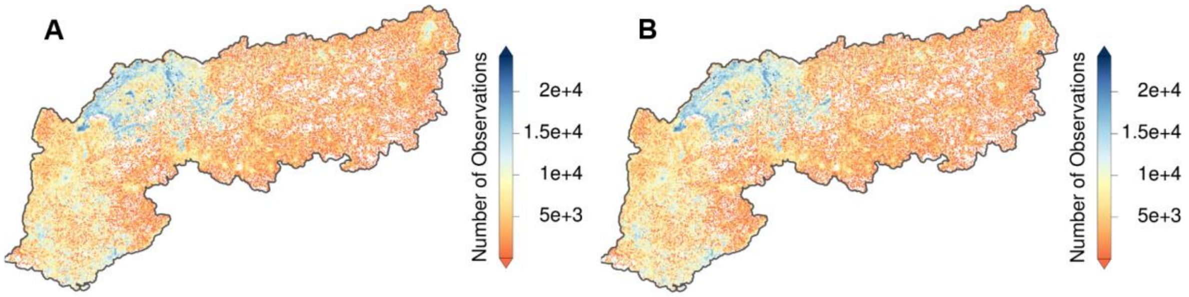

Supplementary Figure 32. Distribution density of our original (a) and refined species observational dataset (b) across the extended European Alps. (**A**) shows the target group observation density of the original observational dataset (i.e., 6'655'163 observations accurate to 11.1 meters for 4’250 species). (**B**) shows the observation density of our refined observational dataset (6'603'305 unique observations for 3’167 species), i.e., the whole species observations used for model calibration. Distribution of density was aggregated at 2 km resolution for better visual representation and log transformed.

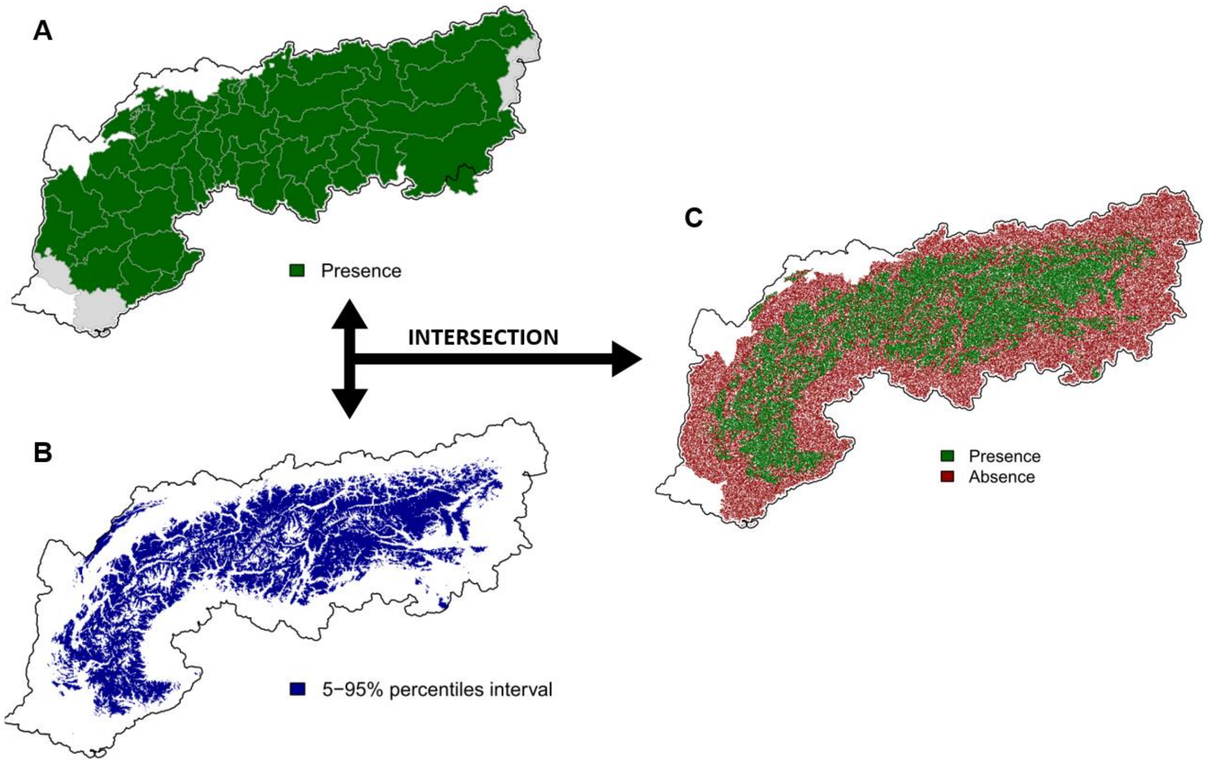

Supplementary Figure 33. Methodological framework used to generate the FA independent test dataset at 100 m resolution. Here is presented an example for *Soldanella alpina*. The initial FA raw distribution of *Soldanella alpina* (**A**) is summarized within 54 expert-based native political units (green is regional presence) [1] and was intersected with the 5-95% percentiles elevation interval of *Soldanella alpina* (**B**). This interval here corresponds of an elevational range of ~1'016 - 2'453 m. It was calculated by extracting values of the DEM over Europe (EU-DEM; https://www.eea.europa.eu/data-and-maps/data/eu-dem), aggregated to 100 x 100 m resolution, with the 8'841 species observations. Final output was a refined independent binary distribution of *Soldanella alpina* from which we inferred 100 m presences and absences, sampled n times afterwards for independent evaluation (**C**). Here, as an example, 100'000 presences and absences were plotted respectively.

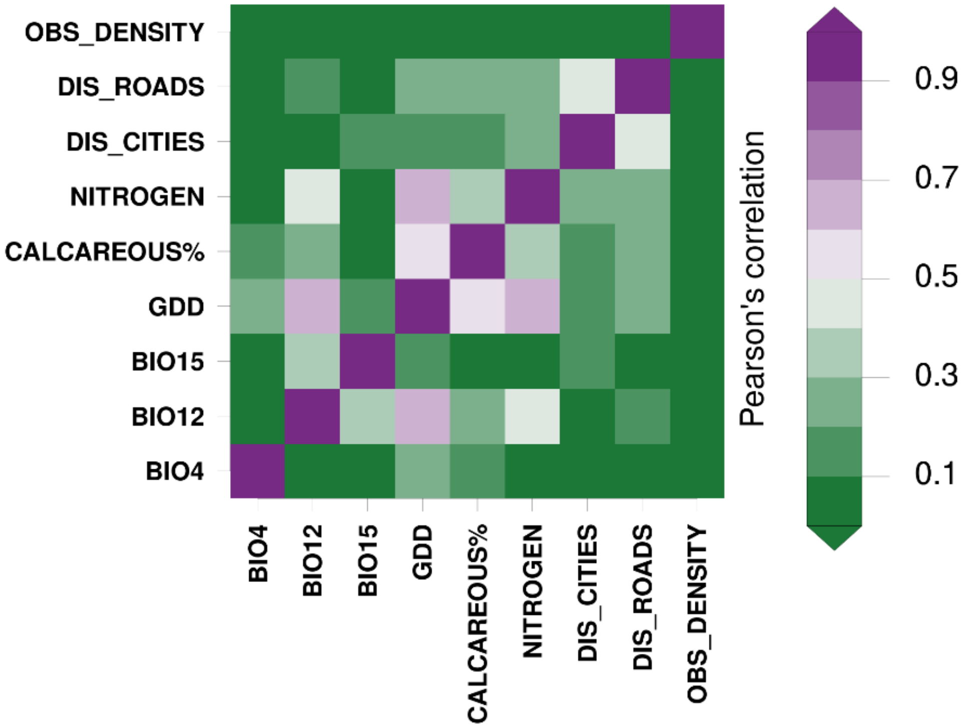

Supplementary Figure 34. Pearson correlation tests between every environmental predictor (except categorical land cover) and bias covariate considered in our models. Climate included growing degree days (GDD), annual precipitation (BIO12), temperature (BIO4) and precipitation seasonality (BIO15). Soil included soil nitrogen (NITROGEN) and substrate composition (CALCAREOUS%). The bias covariates included observation density (OBS_DENSITY), distance to roads (DIS_ROADS) and cities (DIS_CITIES).

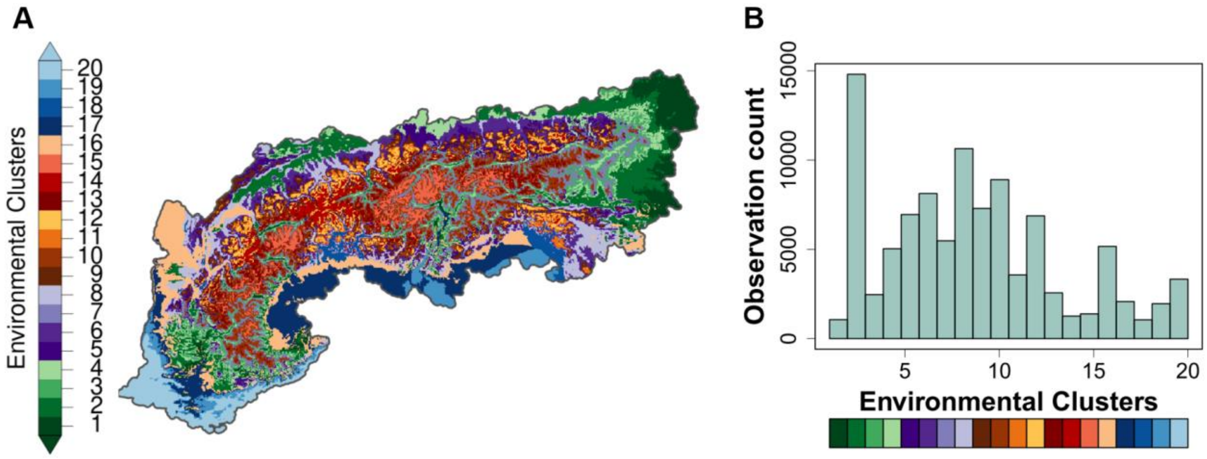

Supplementary Figure 35. Environmental cluster distribution and frequency in observations across the study region. (**A**) describes the distribution of the 20 environmental clusters over the study area obtained with the R function *wsl.ebc* [13]. Those were obtained at 100 m resolution based on our four climate and two soil predictors. (**B**) summarizes the species observation frequencies per environmental cluster regarding the refined observational dataset. Here, 100’000 species observations were sampled randomly without replacements over the cluster map. We see that the sampling design of our refined observational dataset is environmentally biased towards cluster 2 and clusters 5 to 12, i.e., the environmental space of Switzerland (~50% of the refined observational dataset).

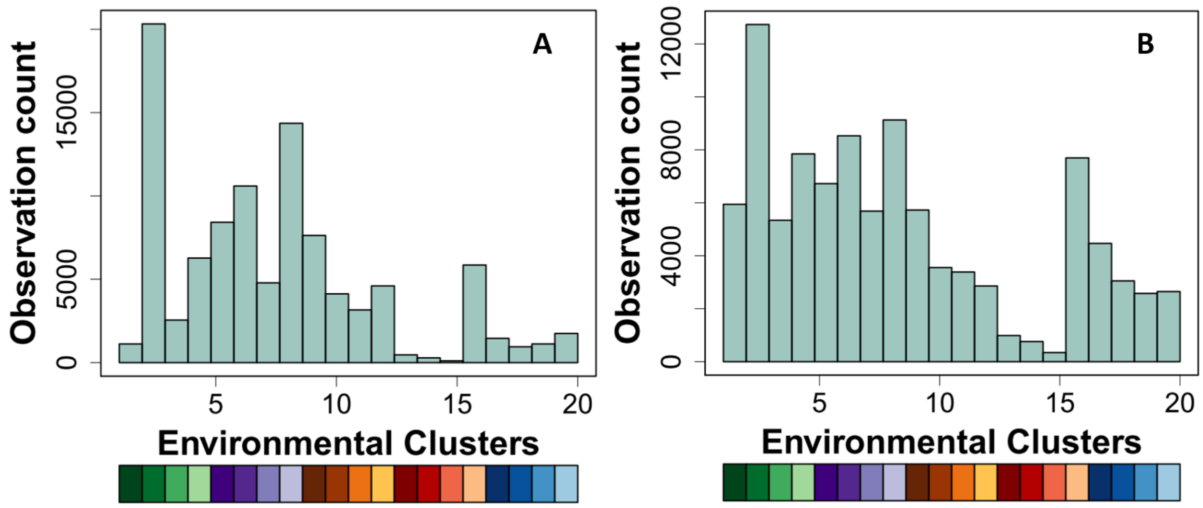

Supplementary Figure 36. Species observation frequencies per environmental cluster before (**A**) and after EBC (**B**) for the 1248 corrected species across the study region. Here, 100'000 non-corrected (**A**) and corrected species observations (**B**) were sampled randomly without replacements over the cluster map. We see that the sampling design in (**A**) is, as the refined observational dataset, environmentally biased towards cluster 2 and clusters 5 to 12, whereas in (**B**), observations frequencies were balanced across environmental clusters based on their respective log(area).

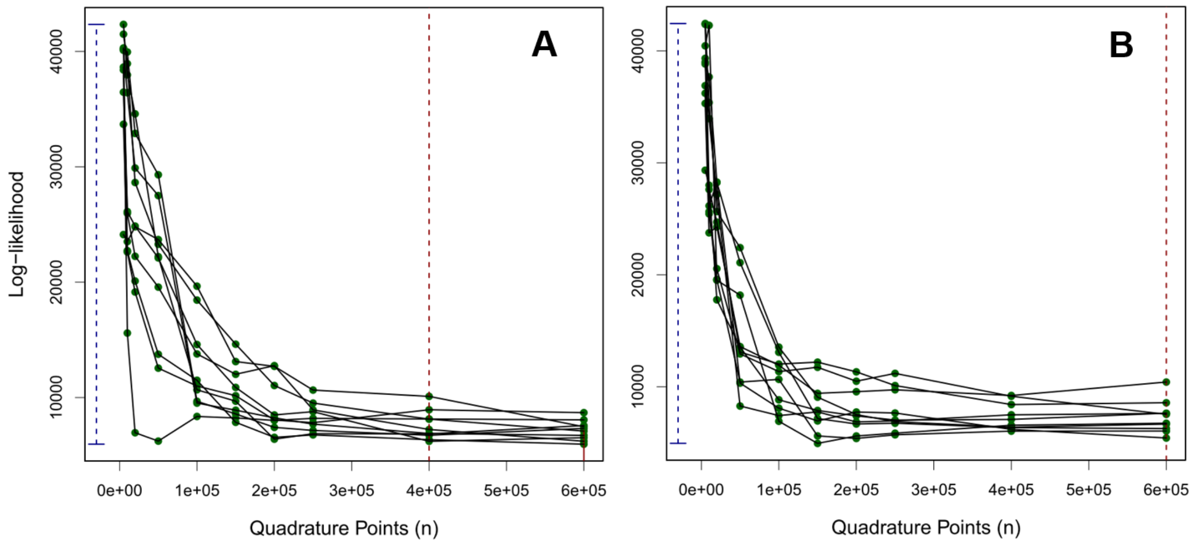

Supplementary Figure 37. An example of PPM log-likelihood convergence for *Soldanella alpina*. Here are shown the PPM convergence tests for models including as land cover the ECOCHANGE (**A**) and HERCULES categories (**B**). To obtain the adequate number of quadrature points, convergence tests were applied before model calibration. For this, 10 preliminary repeated series of DWPR were run, each one gradually increasing the number of randomly sampled quadrature points (i.e., 5000, 10’000, 20’000, 50’000, 100’000, 150’000, 200’000, 250’000, 400’000, 600’000). Below solid red line indicates number of quadrature points chosen by the test (e.g., in a, n = 600’000); i.e., position on the x axis where standard deviation of the log-likelihood values < 10% of the standard deviation found in x = 5000. Dotted blue line indicates range of log-likelihood values for x = 5000. Dotted red line indicates the last position on the x axis where standard deviation of the log-likelihood values > 10% of that of found in x = 5000. Note that if no solid red line appeared (e.g. in **B**), a high number of quadrature points (n = 1’000’000) was chosen by default following [13].

A

```
   Test of normality and Homoscedasticity:
  -------------------------------------------

Hawkins Test:

   P-value for the Hawkins test of normality and homoscedasticity:  0

   Either the test of multivariate normality or homoscedasticity (or both) is rejected.
   Provided that normality can be assumed, the hypothesis of MCAR is
   rejected at 0.05 significance level.

Non-Parametric Test:

   P-value for the non-parametric test of homoscedasticity:  5.812039e-13

   Hypothesis of MCAR is rejected at  0.05 significance level.
   The multivariate normality test is inconclusive.
```

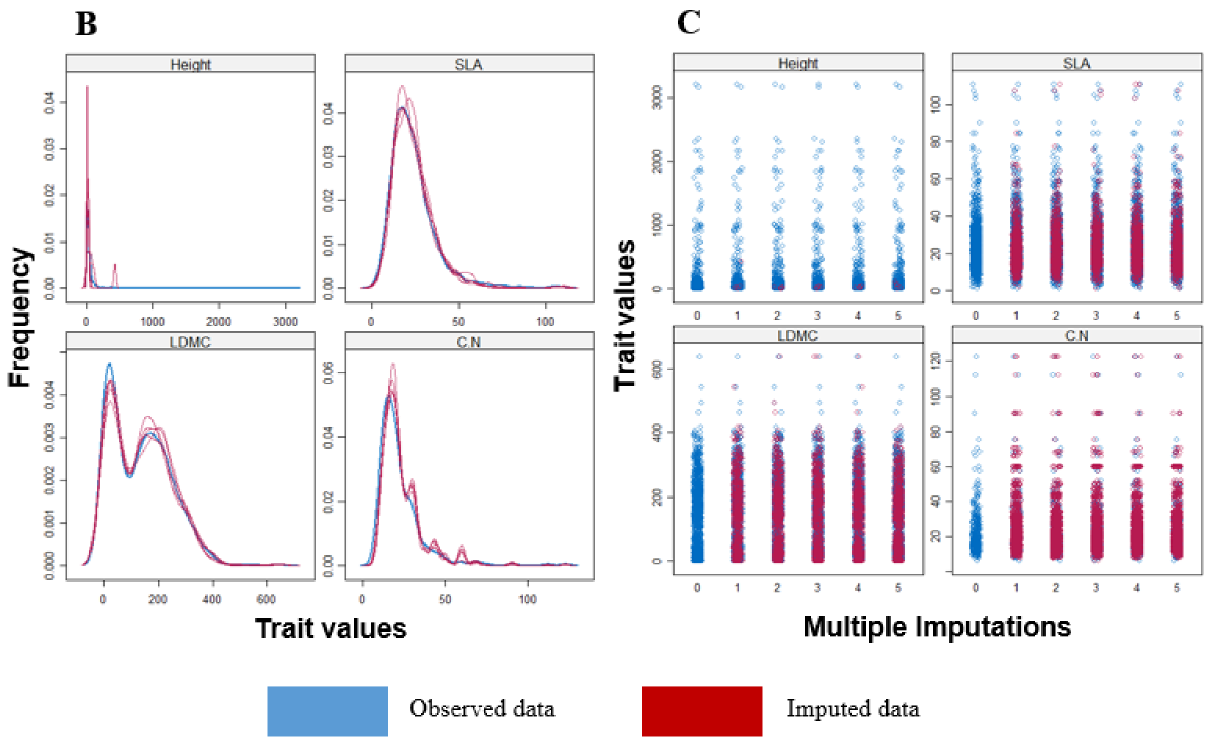

Supplementary Figure 38. Preliminary MCAR (**A**) and multiple-imputation tests (**B**, **C**). MCAR test was run with the *TestMCARNormality* function (*MissMech* R package) [43]. Multiple imputation tests were run 5 times with the *mice* function (*mice* R package) [44] using the random forest method [6]. (**A**) shows R output of the *TestMCARNormality* function. MCAR-test (one-sided) is negative with *p-value* > 0.05. Missing trait values are therefore not missing completely at random. (**B**) shows the distribution frequency plot of four trait values (Height, SLA - specific leaf area, LDMC - leaf dry matter content and C: N - carbon to nitrogen ratio). Blue line represents the distribution of observed trait values whereas the five red lines represent the distribution of imputed values from each five multiple-imputations. (**C**) follows same colour legend and shows same distribution as individual points (or Stripplot). The very similar distributions between observed and imputed data found here gives strong evidences that missing trait values follow a MAR assumption [44].

Supplementary Table 1. Description of extinct species for current and future scenarios. Information on their respective elevation class, soil substrate and vegetation type are also given.

SSP	Timeline	Dispersal	Extinct Species	Elevation Class	Substrate	Type
	Current					
SSP245	2050	Limited				
SSP245	2050	Unlimited				
SSP245	2050	No				
SSP245	2080	Limited	*Antirrhinum latifolium*	Montane	Calcareous	Herbaceous
SSP245	2080	Unlimited	*Antirrhinum latifolium*	Montane	Calcareous	Herbaceous
SSP245	2080	No	*Antirrhinum latifolium*	Montane	Calcareous	Herbaceous
SSP585	2050	Limited	*Iberis saxatilis*	Sub-alpine	Calcareous	Herbaceous
SSP585	2050	Unlimited	*Iberis saxatilis*	Sub-alpine	Calcareous	Herbaceous
SSP585	2050	No	*Iberis saxatilis*	Sub-alpine	Calcareous	Herbaceous
SSP585	2050	Limited	*Antirrhinum latifolium, Iberis saxatilis*			
SSP585	2050	Unlimited	*Antirrhinum latifolium, Iberis saxatilis*			
SSP585	2050	No	*Antirrhinum latifolium, Iberis saxatilis*			

Supplementary Table 2. Complete list of sources of species observations.

Acronyms	Sources	Observations Count	Contribution (%)
AAAAA	Research project	18903	0,245316177942023
ACBOG	Research project	2	2,595526402603e-05
AIPHY	Research project	686	0,0089026555609283
ALOBO	Research project	3	3,8932896039045e-05
ANSES	Research project	43	0,000558038176559645
ASTER	flore-haute-savoie-asters.com	1303	0,0169098545129586
ATFHA	Research project	362	0,00469790278871143
AVENI	Research project	57	0,000739725024741855
BDMZ7	biodiversitymonitoring.ch	96186	1,24826651280386
BDONF	Research project	7393	0,09594363347222
BIOTO	Research project	42	0,00054506054454663
BDMZ9	biodiversitymonitoring.ch	34265	0,444678560925959
CAT07	Research project	7488	0,0971765085134564
CBNA	cbn-alpin.fr	206809	2,68389109897962
CBNMED	cbnmed.fr	431188	5,60353530577767
CENP	Research project	118	0,00153136057753577
Cerabo	Research project	374	0,00485363437286761
CHLAU	Research project	1703	0,0221009073181646
CINCL	Research project	4	5,191052805206e-05
DISEQUALP	klimafonds.gv.at	30289	0,393079496042212
ECODI	Research project	9063	0,117616278933955
ECOME	Research project	281	0,00364671459565722
ECOSP	Research project	1165	0,0151189412951625
EHB	Research project	11772	0,152772684057213
ENGRE	Research project	1104	0,0143273057423686
ESITP	Research project	34	0,00044123948844251
EVINE	Research project	128	0,00166113689766592
FLAVI	Research project	468	0,00607353178209102
FRAPN	Research project	14	0,00018168684818221
GBIF	gbif.org	2242347	29,1003542114882
HERZO	Research project	2	2,595526402603e-05
INFLO	Research project	1692	0,0219581533660214
InfoFlora	infoflora.ch	4027105	52,2622867677728
JORDE	Research project	86	0,00111607635311929
KarstData	Research project	26900	0,349098301150104
LAVAN	Research project	62	0,00080461318480693
LEPJH	Research project	102	0,00132371846532753
LOPAR	Research project	6	7,786579207809e-05
MNHN	mnhn.fr	51	0,000661859232663765
NAENV	Research project	28	0,00036337369636442
NEOT	Research project	60	0,000778657920780901
ONF	onf.fr	4738	0,0614880204776651
PNE	Research project	14713	0,19093989980749

PNM	Research project	335	0,00434750672436003
PNRLU	Research project	647	0,00839652791242071
PNRMB	Research project	537	0,00696898839098906
PNRQ	Research project	371	0,00481470147682857
PNV	Research project	238	0,00308867641909757
RECAL	Research project	10	0,00012977632013015
RESBO	Research project	2847	0,0369473183410537
RESCB	Research project	654	0,00848737133651182
RNLAV	Research project	171	0,00221917507422557
RNPLA	Research project	1	1,2977632013015e-05
SAGEE	Research project	32	0,00041528422441648
SBCOU	Research project	21	0,000272530272273315
SBDRO	Research project	37	0,000480172384481555
SBF	Research project	242	0,00314058694714963
SBNF	Research project	193	0,0025046829785119
SDETB	Research project	239	0,00310165405111059
SERAN	Research project	62	0,00080461318480693
SHNPM	Research project	12	0,00015573158415618
SIGDA	Research project	60	0,000778657920780901
SLL	Research project	1	1,2977632013015e-05
TUXEN	Research project	10	0,00012977632013015
TWW	bafu.admin.ch	419115	5,43912024113478
UAMSJ	Research project	3057	0,0396726210637869
UCLBL	Research project	12	0,00015573158415618
UCOMA	Research project	104	0,00134967372935356
UNBES	Research project	2336	0,0303157483824031
UNDST	Research project	126	0,00163518163363989
UNJOF	Research project	14443	0,187435939163976
UNPRO	Research project	1479	0,0191939177472492
UPAXI	Research project	3827	0,0496653977138084
WILLNER	Research project	52125	0,676459068678407
X	Research project	8	0,00010382105610412
ZER	Monitoring program	22475	0,291672279492512

Supplementary Table 3. Statistics of the original (4’250 species) and refined observational dataset (3’167 species).

	Original dataset	*Refined dataset*
Minimum	1	30
1st Quantile	41	199
Median	305	607
Mean	1566	2085
3rd Quantile	1368	2123
Maximum	42948	42948

Supplementary Table 4. List of predictors used in the EIV mapping. The description and the source of each predictor is provided.

Layer name	Abbreviation	Description	Sources of the layers
DEM_laea_100m	DEM	Digital elevation model	https://www.eea.europa.eu/data-and-maps/data/copernicus-land-monitoring-service-eu-dem
DEM_aspect_laea_100m	Aspect	Aspect	This manuscript, using function *terrain* and R 4.1
DEM_TPI_laea_100m	TPI	Topographic position index	This manuscript, using function *terrain* and R 4.1
DEM_TRI_laea_100m	TRI	Topographic roughness index	This manuscript, using function *terrain* and R 4.1
DEM_TWI_SAGA_laea_100m	TWI	Topographic wetness index	This manuscript, using SAGA-GIS 7.2.0
OPENSTREET_h20_dis_laea_100m	H20	Distance to water	https://www.openstreetmap.org/#map=11/47.0056/7.9740 - Layer calculated with R 4.1, python 3.9 and GDAL/OGR 3.8.0
GLIM_bedrock_types_laea_100m	Geology	Soil geology reclassified in three groups (gradient of CaCO3)	[61] based on [62]

Supplementary Table 5. Spearman rank correlations between observed and predicted EIVs based on repeated split-sampling (Split) and five-fold block cross-validation (CV) tests in the European Alps. EIV-R = soil pH. EIV-N = soil nitrogen, EIV-F = soil moisture.

EIV	Split		CV	
	r		r	
	Mean	SD	Mean	SD
EIV-R	0.843	0.001	0.835	0.029
EIV-N	0.867	0.001	0.852	0.032
EIV-G	0.825	0.002	0.819	0.043
EIV-F	0.860	0.003	0.853	0.027

Supplementary Table 6. Reclassified VOLANTES-HERCULES land cover from 16 to 10 classes.

HERCULES land cover	***Reclassified land cover***
Built-up area	Built-up area
Arable land (non-irrigated)	Arable land (non-irrigated)
Pasture	Pasture
Semi-Natural vegetation	Semi-Natural vegetation
Irrigated arable land	Irrigated arable land
Permanent crops	Permanent crops
Forest	Forest
Sparsely vegetated areas	Others
Beaches, dunes and sands	Others
Inland wetlands	Others
Glaciers and snow	Others
Heather and moorlands	Others
Salines	Removed
Water and coastal flats	Removed

Supplementary Table 7. Complete list of MigClim parameters based on the literature. Parameters in orange were species-specific.

Parameter	Description	Literature
dispKernel	Considers a vector **Pdisp(x)** of dispersal probabilities calculated with a negative exponential dispersal Kernel as in [63,64]: $$Pdisp(x) = e^{-x/\theta} \quad \text{[eqn 21]}$$ where ***x*** is a vector of distances ranging from 100 meters (study resolution) to the maximum dispersal distance of the species in meters with a 100 meters' step, and ***θ*** the minimum dispersal distance of the species in meters. Both ***x*** and ***θ*** were extracted from compiled literature [65,66].	63–66
barrier	Considered a binary raster of physical barriers that was specific to each species habitat (information extracted and compiled from expert data[1,65,67,68]). Based on CORINE land cover 2000 [69], barriers were defined if the feature's area > 2 km^2. In total, three barrier layers were generated, i.e., for species subsisting in (1) open, (2) mixte and (3) forest habitats. Although all layers had common barriers (water, snow, and glaciers), layer (1) additionally included forests, while layer (3) included every land cover class except forests. It is important to note that setting up barriers also allows SDM maps to be filtered according to each species preferred habitat, thus making spatial predictions more realistic.	1,65,67–69
barrierType	Defined as 'weak' (i.e., dispersal through pixel corners allowed). Technical parameter that slightly 'eases' species dispersal. Set as default for all species.	
lddFreq	Defines a frequency percentage of long-distance dispersal (LDD) events. It was set to 0.01 (or 1%) as in [70,71]. Information per species unknown as these events are known to be very stochastic in nature [72].	70,71
lddMinDist	Defines the LDD minimum distance. It was automatically set following the minimum value recommendation of the *MigClim* user guide [73].	73
lddMaxDist	(f) Defines the LDD maximum distance. It was set according to each dispersal distance categories following [71].	71
iniMatAge	Stands for 'initial maturity age'. It was set to 2, which means that colonized cells may produce new propagules only after 2 model dispersal steps (or 2 years, i.e., once the plant hypothetically reached initial maturity). This choice was driven by the variety of vegetation type in our dataset.	
propaguleProd	Defines the probability of propagule production for each age between the initial maturity age and full maturity. It was set to a vector defined as c(0.01, 0.08, 0.5, 0.92), i.e. a simple sigmoid function generally known to represent plant growth [73,74].	73,74
replicateNb	Defines the number of *MigClim* simulations. This was set to 3 to account for model stochasticity as recommended in [71,73].	71,73

Supplementary Table 8. Complete list of sources of trait data

SOURCES	NUMBER OF SAMPLES	CONTRIBUTION (%)
ARVES 2012	7635	4.48906397
BISON	1300	0.76434619
BRZEZIECKI & KIENAST, 1994	26	0.015286924
CBNA	9365	5.506232361
CHOLER	196	0.115239887
COLACE	779	0.458019755
CORNELISSEN, 1999	48	0.028222013
CORNELISSEN, UNPUBLISHED DATA	115	0.06761524
DISEQUALP	5349	3.144990593
ECOCHANGE	498	0.292803387
EQUIPE TDE	11825	6.952610536
FIFTH	28765	16.91262935
FLORA ALPINA	248	0.145813735
FLORA OF ESTONIAN NSV	1166	0.685559737
HYDROTRDB	4735	2.783984008
INTRABIODIV	2522	1.482831609
KEW SEED INFORMATION DATABASE	193	0.113476011
LEDA	11001	6.468132643
LESTRUECSPE	12381	7.279515522
MOUVE	668	0.39275635
MSS	1351	0.794332079
NIINEMETS AND KULL, 2003	60	0.035277516
ODYSSEE	5882	3.458372531
ODYSSEE	155	0.091133584
OLO	700	0.411571025
ORIGINALPS	31650	18.60888993
SCHWEINGRUBER	3199	1.880879586
STACE, 1991	82	0.048212606
TTT_ALPS	3795	2.231302916
TTT_NON_ALPS	23684	13.92521167
VAN DER MEIJDEN ET AL., 1990	74	0.043508937
VITTOZ&ENGLER	508	0.298682973
WILLNER	125	0.073494826

Supplementary Table 9. Chosen Zonation parameters

Zonation setting file (.dat)	Zonation feature list file (.ssp)
removal rule = 1 (CAZ) or 2 (ABF)	*weight* = sp PU[1] + sp FU[2]
warp factor = 100 [75]	*alpha* = 1 (default)
use mask = 0 (selection) or 1 (expansion)	*bqp* = 1 (default)
mask file = I-II-2000_IUCN.tif	*bqp_p* = 1 (default)
	cellrem = 0.25 [75]

[1] Phylogenetic Uniqueness

[2] Functional Uniqueness

Parameters in blue were chosen by default, i.e., connectivity settings off.

Supplementary Table 10. Description of the 26 SCP maps generated for each CAZ and ABF algorithm (i.e., 52 maps of prioritisation in total)

	**Year**	**Emissions**	**Dispersal**	**IUCN I-II reserves + Natura 2000**
1	Current			Yes
2	Current			No
3	2050	SSP245	no dispersal	Yes
4	2050	SSP245	no dispersal	No
5	2050	SSP245	unlimited dispersal	Yes
6	2050	SSP245	unlimited dispersal	No
7	2050	SSP245	limited dispersal	Yes
8	2050	SSP245	limited dispersal	No
9	2050	SSP585	no dispersal	Yes
10	2050	SSP585	no dispersal	No
11	2050	SSP585	unlimited dispersal	Yes
12	2050	SSP585	unlimited dispersal	No
13	2050	SSP585	limited dispersal	Yes
14	2050	SSP585	limited dispersal	No
15	2080	SSP245	no dispersal	Yes
16	2080	SSP245	no dispersal	No
17	2080	SSP245	unlimited dispersal	Yes
18	2080	SSP245	unlimited dispersal	No
19	2080	SSP245	limited dispersal	Yes
20	2080	SSP245	limited dispersal	No
21	2080	SSP585	no dispersal	Yes
22	2080	SSP585	no dispersal	No
23	2080	SSP585	unlimited dispersal	Yes
24	2080	SSP585	unlimited dispersal	No
25	2080	SSP585	limited dispersal	Yes
26	2080	SSP585	limited dispersal	No

References:

1. Aeschimann, D., Lauber, K., Moser, D. M. & Theurillat, J. P. *Flora alpina: ein Atlas sämtlicher 4500 Gefässpflanzen der Alpen.* (Haupt, 2004).
2. Chauvier, Y. *et al.* gbif.range - An R package to generate species range maps based on ecoregions and a user-friendly GBIF wrapper. R package version 1.1. *Envidat* Preprint at https://doi.org/10.16904/envidat.352 (2022).
3. Zizka, A. *et al.* CoordinateCleaner: Standardized cleaning of occurrence records from biological collection databases. *Methods Ecol Evol* **10**, 744–751 (2019).
4. Descombes, P. *et al.* Spatial modelling of ecological indicator values improves predictions of plant distributions in complex landscapes. *Ecography* **43**, 1448–1463 (2020).
5. Landolt, E. *et al. Flora indicativa: Okologische Zeigerwerte und biologische Kennzeichen zur Flora der Schweiz und der Alpen.* (Haupt., 2010).
6. Breiman, L. Random Forests. *Mach Learn* **45**, 5–32 (2001).
7. Roberts, D. R. *et al.* Cross-validation strategies for data with temporal, spatial, hierarchical, or phylogenetic structure. *Ecography* **40**, 913–929 (2017).
8. Chauvier, Y. *et al.* Resolution in species distribution models shapes spatial patterns of plant multifaceted diversity. *Ecography* **2022**, e05973 (2022).
9. Mohler, C. L. Effect of sampling pattern on estimation of species distributions along gradients. *Vegetatio* **54**, 97–102 (1983).
10. Austin, M. P. & Heyligers, P. C. Vegetation survey design for conservation: Gradsect sampling of forests in North-eastern New South Wales. *Biol Conserv* **50**, 13–32 (1989).
11. Hirzel, A. & Guisan, A. Which is the optimal sampling strategy for habitat suitability modelling. *Ecol Modell* **157**, 331–341 (2002).
12. Albert, C. H. *et al.* Sampling in ecology and evolution - bridging the gap between theory and practice. *Ecography* **33**, 1028–1037 (2010).
13. Chauvier, Y. *et al.* Novel methods to correct for observer and sampling bias in presence-only species distribution models. *Global Ecology and Biogeography* **30**, 2312–2325 (2021).
14. Brock, G., Pihur, V., Datta, S. & Datta, S. clValid, an R package for cluster validation. *J Stat Softw* (2011) doi:10.18637/jss.v025.i04.
15. Renner, I. W. *et al.* Point process models for presence-only analysis. *Methods Ecol Evol* **6**, 366–379 (2015).
16. Warton, D. I. & Shepherd, L. C. Poisson point process models solve the ‘pseudo-absence problem’ for presence-only data in ecology. *Annals of Applied Statistics* **4**, 1383–1402 (2010).
17. Renner, I. W. & Warton, D. I. Equivalence of MAXENT and Poisson Point Process Models for Species Distribution Modeling in Ecology. *Biometrics* **69**, 274–281 (2013).
18. Descombes, P. *et al.* Strategies for sampling pseudo-absences for species distribution models in complex mountainous terrain. *bioRxiv* (2022) doi:https://doi.org/10.1101/2022.03.24.485693.
19. Barbet-Massin, M., Jiguet, F., Albert, C. H. & Thuiller, W. Selecting pseudo-absences for species distribution models: How, where and how many? *Methods Ecol Evol* **3**, 327–338 (2012).
20. Cressie, N. A. C. *Statistics for spatial data*. (John Wiley & Sons., 1993). doi:10.1002/9781119115151.
21. McCullagh, P. Generalized linear models. *Eur J Oper Res* **16**, 285–292 (1984).

22. Zou, H. & Hastie, T. Regression and variable selection via the elastic net. *J R Stat Soc Series B Stat Methodol* **67**, 301–320 (2005).
23. Friedman, J., Hastie, T. & Tibshirani, R. Regularization Paths for Generalized Linear Models via Coordinate Descent. *J Stat Softw* **33**, 1–22 (2010).
24. Friedman, J., Hastie, T. & Tibshirani, R. glmnet: Lasso and elastic-net regularized generalized linear models. R package version 4.1. *CRAN* Preprint at https://CRAN.R-project.org/package=glmnet (2021).
25. Hill, M. O. Diversity and Evenness: A Unifying Notation and Its Consequences. *Ecology* **54**, 427–432 (1973).
26. Li, D. hillR: taxonomic, functional, and phylogenetic diversity and similarity through Hill Numbers. *The Journal of Open Source Software* **3**, 1041 (2018).
27. Jin, Y. & Qian, H. V.PhyloMaker: an R package that can generate very large phylogenies for vascular plants. *Ecography* **42**, 1353–1359 (2019).
28. Zanne, A. E. *et al.* Three keys to the radiation of angiosperms into freezing environments. *Nature* **506**, 89–92 (2014).
29. Smith, S. A. & Brown, J. W. Constructing a broadly inclusive seed plant phylogeny. *Am J Bot* **105**, 302–314 (2018).
30. Chao, A., Chiu, C. H. & Jost, L. Unifying species diversity, phylogenetic diversity, functional diversity, and related similarity and differentiation measures through hill numbers. *Annu Rev Ecol Evol Syst* **45**, 297–324 (2014).
31. Allen, B., Kon, M. & Bar-Yam, Y. A new phylogenetic diversity measure generalizing the shannon index and its application to phyllostomid bats. *American Naturalist* **174**, 236–243 (2009).
32. Devictor, V. *et al.* Spatial mismatch and congruence between taxonomic, phylogenetic and functional diversity: The need for integrative conservation strategies in a changing world. *Ecol Lett* **13**, 1030–1040 (2010).
33. Forest, F. *et al.* Preserving the evolutionary potential of floras in biodiversity hotspots. *Nature* **445**, 757–760 (2007).
34. Pavoine, S., Gasc, A., Bonsall, M. B. & Mason, N. W. H. Correlations between phylogenetic and functional diversity: Mathematical artefacts or true ecological and evolutionary processes? *Journal of Vegetation Science* **24**, 781–793 (2013).
35. Pardo, I. *et al.* Spatial congruence between taxonomic, phylogenetic and functional hotspots: true pattern or methodological artefact? *Divers Distrib* **23**, 209–220 (2017).
36. Thuiller, W. *et al.* Productivity begets less phylogenetic diversity but higher uniqueness than expected. *J Biogeogr* **47**, 44–58 (2020).
37. Májeková, M. *et al.* Evaluating Functional diversity: Missing trait data and the importance of species abundance structure and data transformation. *PLoS One* **11**, e0152532 (2016).
38. Nakagawa, S. & Freckleton, R. P. Missing inaction: the dangers of ignoring missing data. *Trends Ecol Evol* **23**, 592–596 (2008).
39. Penone, C. *et al.* Imputation of missing data in life-history trait datasets: Which approach performs the best? *Methods Ecol Evol* **5**, 1–10 (2014).
40. Johnson, T. F., Isaac, N. J. B., Paviolo, A. & González-Suárez, M. Handling missing values in trait data. *Global Ecology and Biogeography* **00**, 1–12 (2020).
41. Taugourdeau, S., Villerd, J., Plantureux, S., Huguenin-Elie, O. & Amiaud, B. Filling the gap in functional trait databases: Use of ecological hypotheses to replace missing data. *Ecol Evol* **4**, 944–958 (2014).

42. Madley-Dowd, P., Hughes, R., Tilling, K. & Heron, J. The proportion of missing data should not be used to guide decisions on multiple imputation. *J Clin Epidemiol* **110**, 63–73 (2019).
43. Jamshidian, M., Jalal, S. & Jansen, C. Missmech: An R package for testing homoscedasticity, multivariate normality, and missing completely at random (MCAR). *J Stat Softw* **56**, 1–31 (2014).
44. van Buuren, S. & Groothuis-Oudshoorn, K. mice: Multivariate imputation by chained equations in R. *J Stat Softw* **45**, 1–67 (2011).
45. Pavoine, S., Vallet, J., Dufour, A.-B., Gachet, S. & Daniel, H. On the challenge of treating various types of variables: application for improving the measurement of functional diversity. *Oikos* **118**, 391–402 (2009).
46. Maire, E., Grenouillet, G., Brosse, S. & Villéger, S. How many dimensions are needed to accurately assess functional diversity? A pragmatic approach for assessing the quality of functional spaces. *Global Ecology and Biogeography* **24**, 728–740 (2015).
47. Maechler, M., Rousseeuw, P., Struyf, A., Hubert, M. & Hornik, K. Cluster: cluster analysis basics and extensions. R package version 2.1.2. *CRAN* (2021).
48. Mouchet, M. *et al.* Towards a consensus for calculating dendrogram-based functional diversity indices. *Oikos* **117**, 794–800 (2008).
49. Oksanen, J. *et al.* vegan: community ecology package. R package version 2.4-2. *CRAN* (2017).
50. Chiu, C. H. & Chao, A. Distance-based functional diversity measures and their decomposition: A framework based on hill numbers. *PLoS One* **9**, (2014).
51. Walker, B., Kinzig, A. & Langridge, J. Plant Attribute Diversity, Resilience, and Ecosystem Function: The Nature and Significance of Dominant and Minor Species. *Ecosystems* **2**, 95–113 (1999).
52. Guiasu, R. C. & Guiasu, S. The weighted Gini-Simpson index: Revitalizing an old index of biodiversity. *International Journal of Ecology* **2012**, 478728 (2012).
53. Crisp, M. D., Laffan, S., Linder, H. P. & Monro, A. Endemism in the Australian flora. *J Biogeogr* **28**, 183–198 (2001).
54. Daru, B. H., Farooq, H., Antonelli, A. & Faurby, S. Endemism patterns are scale dependent. *Nat Commun* **11**, 2115 (2020).
55. Rosauer, D., Laffan, S. W., Crisp, M. D., Donnellan, S. C. & Cook, L. G. Phylogenetic endemism: A new approach for identifying geographical concentrations of evolutionary history. *Mol Ecol* **18**, 4061–4072 (2009).
56. Grenié, M., Denelle, P., Tucker, C. M., Munoz, F. & Violle, C. funrar: An R package to characterize functional rarity. *Divers Distrib* **23**, 1365–1371 (2017).
57. Violle, C. *et al.* Functional rarity: the ecology of outliers. *Trends Ecol Evol* **32**, 356–367 (2017).
58. Grenié, M. *et al.* Functional rarity of coral reef fishes at the global scale: Hotspots and challenges for conservation. *Biol Conserv* **226**, 288–299 (2018).
59. Pollock, L. J. *et al.* Phylogenetic diversity meets conservation policy: small areas are key to preserving eucalypt lineages. *Philosophical Transactions of the Royal Society B: Biological Sciences* **370**, 20140007–20140007 (2015).
60. Pollock, L. J., Thuiller, W. & Jetz, W. Large conservation gains possible for global biodiversity facets. *Nature* **546**, 141–144 (2017).
61. Chauvier, Y. *et al.* Influence of climate, soil, and land cover on plant species distribution in the European Alps. *Ecol Monogr* **91**, e01433 (2021).

62. Hartmann, J. & Moosdorf, N. The new global lithological map database GLiM: A representation of rock properties at the Earth surface. *Geochemistry, Geophysics, Geosystems* **13**, 7 (2012).
63. Sutherland, G. D., Harestad, A. S., Price, K. & Lertzman, K. P. Scaling of natal dispersal distances in terrestrial birds and mammals. *Ecology and Society* **4**, 16 (2000).
64. Zurell, D. *et al.* Benchmarking novel approaches for modelling species range dynamics. *Glob Chang Biol* **22**, 2651–2664 (2016).
65. Lososová, Z. *et al.* Seed dispersal distance classes and dispersal modes for the European flora. *Global Ecology and Biogeography* 1–10 (2023) doi:10.1111/geb.13712.
66. Vittoz, P. & Engler, R. Seed dispersal distances: A typology based on dispersal modes and plant traits. *Botanica Helvetica* **117**, 109–124 (2007).
67. Juillerat, P. *et al. Flora Helvetica Checklist 2017: der Gefässpflanzen der Schweiz = de la flore vasculaire de la Suisse = della flora vascolare della Svizzera*. *Zurich Open Repository and Archive* (Info Flora, 2017). doi:10.5167/uzh-165877.
68. Lauber, K., Wagner, G., Gygax, A., Eggenberg, S., & Michel, A. *Flora helvetica*. (Vienna, Bern, Stuttgart: P. Haupt., 2001).
69. Bossard, M., Feranec, J. & Otahel, J. *CORINE Land Cover technical Guide - Addendum 2000*. *EEA Technical Report* http://www.eea.europa.eu/publications/COR0-landcover (2000).
70. Engler, R. & Guisan, A. MigClim: Predicting plant distribution and dispersal in a changing climate. *Divers Distrib* **15**, 590–601 (2009).
71. Engler, R. *et al.* Predicting future distributions of mountain plants under climate change: Does dispersal capacity matter? *Ecography* **32**, 34–45 (2009).
72. Nathan, R. Long-distance dispersal of plants. *Science (1979)* **313**, 786–788 (2006).
73. Engler, R., Hordijk, W. & Guisan, A. The MIGCLIM R package - seamless integration of dispersal constraints into projections of species distribution models. *Ecography* **35**, 872–878 (2012).
74. Liu, J. H. *et al.* Simulation of crop growth, time to maturity and yield by an improved sigmoidal model. *Sci Rep* **8**, 6–11 (2018).
75. Minin, E. Di, Veach, V., Lehtomäki, J., Pouzols, F. M. & Moilanen, A. *A quick introduction to Zonation, version 1 (for Zv4)*. (Univerity of Helsinki, 2014).
