# Supplementary material for: Transnational conservation to anticipate future plant shifts in Europe
Source: Nat Ecol Evol. 2024 Jan 22;8(3):454–66. doi: 10.1038/s41559-023-02287-3 (PMC10927550; doi:10.1038/s41559-023-02287-3)
Supplement: Supplementary file 2 — Reporting Summary [file 41559_2023_2287_MOESM2_ESM.pdf]

nature portfolio

Corresponding author(s): Yohann Chauvier-Mendes

Last updated by author(s): Jul 10, 2023

Reporting Summary

Nature Portfolio wishes to improve the reproducibility of the work that we publish. This form provides structure for consistency and transparency in reporting. For further information on Nature Portfolio policies, see our Editorial Policies and the Editorial Policy Checklist.

Statistics

For all statistical analyses, confirm that the following items are present in the figure legend, table legend, main text, or Methods section.

n/a	Confirmed	
☐	☒	The exact sample size (*n*) for each experimental group/condition, given as a discrete number and unit of measurement
☒	☐	A statement on whether measurements were taken from distinct samples or whether the same sample was measured repeatedly
☐	☒	The statistical test(s) used AND whether they are one- or two-sided<br>*Only common tests should be described solely by name; describe more complex techniques in the Methods section.*
☐	☒	A description of all covariates tested
☐	☒	A description of any assumptions or corrections, such as tests of normality and adjustment for multiple comparisons
☐	☒	A full description of the statistical parameters including central tendency (e.g. means) or other basic estimates (e.g. regression coefficient) AND variation (e.g. standard deviation) or associated estimates of uncertainty (e.g. confidence intervals)
☐	☒	For null hypothesis testing, the test statistic (e.g. *F*, *t*, *r*) with confidence intervals, effect sizes, degrees of freedom and *P* value noted<br>*Give P values as exact values whenever suitable.*
☒	☐	For Bayesian analysis, information on the choice of priors and Markov chain Monte Carlo settings
☒	☐	For hierarchical and complex designs, identification of the appropriate level for tests and full reporting of outcomes
☐	☒	Estimates of effect sizes (e.g. Cohen's *d*, Pearson's *r*), indicating how they were calculated

Our web collection on statistics for biologists contains articles on many of the points above.

Software and code

Policy information about availability of computer code

Data collection	R 4.1, python 3.9, GDAL/OGR 3.8.0, SAGA-GIS 7.2.0, Zonation 4 (CAZ/ABF)
Data analysis	R 4.1, python 3.9, GDAL/OGR 3.8.0, SAGA-GIS 7.2.0, Zonation 4 (CAZ/ABF), https://github.com/8Ginette8/wsl.biodiv, chelsa-cmip6 v1.0 python library, MigClim v1.6, V.Phylomaker v1.0, hillR v0.5.2, phyloregion v1.0.8, funrar v1.5.0, gbif.range v1.0, clValid v0.7, glmnet v4.1.8, MissMech v1.0.2, mice v3.16.0, cluster v2.1.4, and vegan v2.6.4 R packages.

For manuscripts utilizing custom algorithms or software that are central to the research but not yet described in published literature, software must be made available to editors and reviewers. We strongly encourage code deposition in a community repository (e.g. GitHub). See the Nature Portfolio guidelines for submitting code & software for further information.

Data

Policy information about availability of data

All manuscripts must include a data availability statement. This statement should provide the following information, where applicable:

- Accession codes, unique identifiers, or web links for publicly available datasets
- A description of any restrictions on data availability
- For clinical datasets or third party data, please ensure that the statement adheres to our policy

All data, code, and materials supporting the findings of this study are available in the EnviDat repository https://doi.org/10.16904/envidat.371, which provides options to download single (via WGET and singular FTP links) or every files (S3 access using the software Cyberduck or any other S3 clients) employed in this project.

Human research participants

Policy information about studies involving human research participants and Sex and Gender in Research.

Reporting on sex and gender	We did not use human participants for this study.
Population characteristics	We did not use human participants for this study.
Recruitment	We did not use human participants for this study.
Ethics oversight	We did not use human participants for this study.

Note that full information on the approval of the study protocol must also be provided in the manuscript.

Field-specific reporting

Please select the one below that is the best fit for your research. If you are not sure, read the appropriate sections before making your selection.

☐ Life sciences ☐ Behavioural & social sciences ☒ Ecological, evolutionary & environmental sciences

For a reference copy of the document with all sections, see nature.com/documents/nr-reporting-summary-flat.pdf

Ecological, evolutionary & environmental sciences study design

All studies must disclose on these points even when the disclosure is negative.

Study description	Here, we investigate the efficiency of the current European Alps' PAs network, and of its potential transnational expansion, in protecting the plant multifaceted diversity and uniqueness of this region at present and for the 2050 and 2080 horizons, under biologically informed (realistic) dispersal, two Shared Socioeconomic Pathways (SSPs), seven Global Circulation Model (GCMs) and two land cover (LC) change scenarios. Using an ensemble of species distribution models (SDMs) for 1,711 plant species at 100-m resolution, a high coverage database of species traits and two mega-phylogenies, we predicted future changes in species distributional range, multifaceted diversity and uniqueness in the study area. We then identified conservation priorities for all types of diversity, areas where these priorities are stable into the future (overlap in current and future priorities), and areas that are critical for expanding the current PA network to meet the 2030 targets while achieving a resilient and effective conservation network.
Research sample	(a) Sample (~6 millions) of plant observations (~4'000 species) compiled for the European Alps from many different sources including GBIF (https://doi.org/10.15468/dd.mb6jz) and 76 institutions. (b) Sample of ecological traits (4308 averaged traits) from 33 different institutions. (c) For the phylogeny, see these methods and sources: https://doi.org/10.1111/ecog.04434
Sampling strategy	No sample-size calculation performed because we are here employing opportunistic data (from online sources and various institutions) already sampled.
Data collection	A large compilation of opportunistic/online/institution observations of plants, traits, phylogenies and community plots.
Timing and spatial scale	Plant species observed and/or sampled from 1980 to 2021 accurate to 11.1 meters.
Data exclusions	Observations accurate to > 11.1 meters were excluded.
Reproducibility	All attempts to reproduce the experiments were successful.
Randomization	Not relevant because modelling was used as a main method.
Blinding	Not relevant because a large amount of independent data were compiled.

Did the study involve field work? ☐ Yes ☒ No

Reporting for specific materials, systems and methods

We require information from authors about some types of materials, experimental systems and methods used in many studies. Here, indicate whether each material, system or method listed is relevant to your study. If you are not sure if a list item applies to your research, read the appropriate section before selecting a response.

Materials & experimental systems

n/a	Involved in the study
☒	☐ Antibodies
☒	☐ Eukaryotic cell lines
☒	☐ Palaeontology and archaeology
☒	☐ Animals and other organisms
☒	☐ Clinical data
☒	☐ Dual use research of concern

Methods

n/a	Involved in the study
☒	☐ ChIP-seq
☒	☐ Flow cytometry
☒	☐ MRI-based neuroimaging
